# A method for the generation of human stem cell-derived alpha cells

**DOI:** 10.1038/s41467-020-16049-3

**Published:** 2020-05-07

**Authors:** Quinn P. Peterson, Adrian Veres, Lihua Chen, Michael Q. Slama, Jennifer H. R. Kenty, Shaimaa Hassoun, Matthew R. Brown, Haiqiang Dou, Caden D. Duffy, Quan Zhou, Aleksey V. Matveyenko, Björn Tyrberg, Maria Sörhede-Winzell, Patrik Rorsman, Douglas A. Melton

**Affiliations:** 1 Department of Stem Cell and Regenerative Biology, Harvard University, Cambridge, MA USA; 2 Department of Physiology and Biomedical Engineering, Mayo Clinic, Rochester, MN USA; 3 Center for Regenerative Medicine, Mayo Clinic, Rochester, MN USA; 4 Cardiovascular, Renal and Metabolism and Early Development, BioPharmaceuticals R&D, AstraZeneca, Gothenburg, Sweden; 5 Department of Physiology, University of Gothenburg, Gothenburg, Sweden; 6 Radcliffe Department of Medicine, University of Oxford, Oxford, UK; 7 Present Address: Cardiovascular and Metabolic Diseases, Institut de Recherches Servier, Suresnes, France

**Keywords:** Biological techniques, Stem-cell differentiation, Pancreas

## Abstract

The generation of pancreatic cell types from renewable cell sources holds promise for cell replacement therapies for diabetes. Although most effort has focused on generating pancreatic beta cells, considerable evidence indicates that glucagon secreting alpha cells are critically involved in disease progression and proper glucose control. Here we report on the generation of stem cell-derived human pancreatic alpha (SC-alpha) cells from pluripotent stem cells via a transient pre-alpha cell intermediate. These pre-alpha cells exhibit a transcriptional profile similar to mature alpha cells and although they produce proinsulin protein, they do not secrete significant amounts of processed insulin. Compound screening identified a protein kinase c activator that promotes maturation of pre-alpha cells into SC-alpha cells. The resulting SC-alpha cells do not express insulin, share an ultrastructure similar to cadaveric alpha cells, express and secrete glucagon in response to glucose and some glucagon secretagogues, and elevate blood glucose upon transplantation in mice.

## Abstract (web summary)

Deriving functional pancreatic cell types from human stem cells may have important clinical applications. Building on previous work, here the authors generate stem cell-derived alpha cells via a polyhormonal intermediate, which have a gene expression pattern similar to human islet alpha cells and behave as such when transplanted into mice.

## Introduction

Although diabetes primarily involves beta cell dysfunction, there is mounting evidence that alpha cell defects play a role in disease etiology^1,2^. Patients with type 1 diabetes must cope with significant fluctuation in blood glucose levels including acute hypoglycemia^3^.

In the healthy pancreas, hormone-expressing endocrine cells function within the islets of Langerhans to precisely regulate blood glucose and energy metabolism. During hypoglycemia (low blood glucose), islet alpha cells secrete glucagon^4^ which raises blood glucose levels by increasing glycogenolysis and gluconeogenesis in the liver^5,6^. Although alpha cells persist in diabetic islets, these alpha cells are often incapable of mounting an appropriate glucagon response, perhaps due to the absence of alpha cell−beta cell interactions^7^. Recent studies implicate dysfunction of alpha cells as a contributing factor in the elevated blood glucose levels observed in diabetic patients^4,7^.

Published methods to make pancreatic beta cells^8–11^ all report a minor portion of alpha and delta (somatostatin secreting) cells. Additionally, there are several reports on the conversion of various cell types into alpha cells via transdifferentiation^12–14^. In 2011, Rezania et al.^15^ reported a protocol for generating glucagon-positive cells that exhibited some glucagon secretion in vitro; however, upon transplantation of 1.9 million cells into mice, these cells had limited physiological effects. Despite these early efforts to generate glucagon-positive cells, the production of alpha cells has not been reproduced nor widely adopted by the field.

We previously reported the generation of functional pancreatic beta cells from human pluripotent stem cells using a six-step directed differentiation protocol^8^. That protocol generates beta cells as well as side populations including polyhormonal cells and nonendocrine cells^11^. The presence of polyhormonal cells in pancreatic differentiations have also been observed in other reported protocols^9,16,17^. Several reports described polyhormonal cells as having features of immature beta cells^9,17–19^; however, beyond the expression of insulin protein, there has been little evidence to support the similarity of polyhormonal cells to beta cells, let alone their capacity to differentiate into beta cells. In contrast, others have reported that polyhormonal cells are present during development, contribute to alpha cells later in development, express several markers of alpha cells, and give rise to glucagon-expressing cells when transplanted^11,15,20^.

Reports from the literature have referred to INS+/GCG+ cells in turn as “bi-hormonal”^21^, “polyhormonal”^9,22–24^, “alpha-like cells”^17^, or “alpha cells”^25^. The nomenclature of the INS+/GCG+ cells produced in directed differentiation protocols has become equally confusing. In Veres et al.^11^, we performed extensive scRNA-seq studies on beta cell differentiations and observed a population of cells expressing many markers of alpha cells which we labeled as “alpha-like” cells irrespective of insulin expression. In this manuscript we refine this definition of “alpha-like” cells to distinguish between cells that have detectable insulin protein which we define as “pre-alpha” cells and those that do not express insulin, which we define as “SC-alpha cells”. We favor the term “pre-alpha” rather than “bi-hormonal” or “polyhormonal” because it more accurately depicts the similarity of these cells to alpha cells yet distinguishes them from more mature alpha cells that do not express insulin (SC-alpha).

Here we build upon our previous reports to develop a protocol for the generation of SC-alpha cells. The protocol robustly produces a transient pre-alpha cell intermediate, which has a transcriptional signature similar to alpha cells except that they express insulin in addition to glucagon. We identify a small molecule capable of driving pre-alpha cells to an alpha cell identity and demonstrate the ability to produce approximately 30% SC-alpha cells in vitro. Our results show that these SC-alpha cells have a transcriptional signature similar to human cadaveric alpha cells, are responsive to some glucagon secretagogues, and elicit a robust physiological response within 4 weeks of transplantation in mice. The SC-alpha cells generated can recapitulate central aspects of alpha cell biology and represent an efficient and scalable avenue to produce alpha cells for use in islet organoids for cell replacement therapy, drug screening, disease modeling, and may accelerate exploration of alpha cell biology.

## Results

### Optimization of differentiation to generate pre-alpha cells

**Fig. 1:**
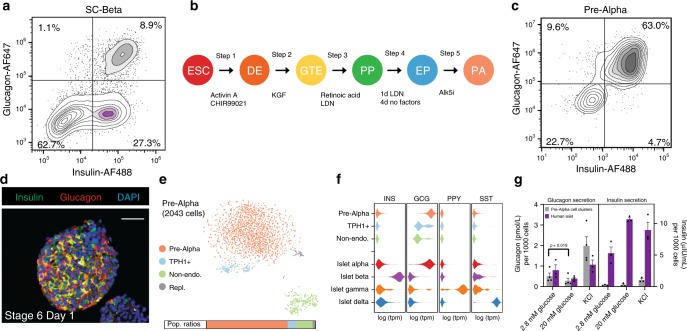
The development of protocols for generating pre-alpha cells. **a** Embryonic stem cell differentiation to SC-beta cells results in a heterogeneous population. In this differentiation, 8.9% of cells coexpress markers for insulin and glucagon. **b** Schematic of directed differentiation protocol from hPSC into pre-alpha cells. The factors added at each step are noted below, catalog numbers are provided in Supplementary Table 1. **c** In the resulting pre-alpha cell differentiation, 63% of the cells stain for glucagon and insulin proteins. **d** Immunofluorescent cross-sectional staining of a cluster for insulin and glucagon. Scale bar = 50 μm. **e** Single-cell RNAseq analysis of pre-alpha cells from the SC-alpha protocol. Expression data are visualized with a tSNE plot where cells are clustered based on the similarity of transcript expression patterns. The expression profile for cells in the pre-alpha cell cluster is similar to the expression profile of human islet alpha cells (Supplementary Fig. 17b). Population ratios by this analysis are consistent with the flow cytometry plot in **c**. **f** Histograms of expression levels (as violin plots) for pancreatic hormones of SC-alpha cells and cadaveric human islets^27^. **g** Pre-alpha cells secrete glucagon, but do not secret insulin. Representative ELISA measurements of secreted glucagon (left panel) from HUES8 differentiated pre-alpha cells or human islets challenged sequentially with 2.8 or 20 mM glucose with a 60-min incubation for each concentration. Representative ELISA measurement of secreted full processed human insulin (right panel) from HUES8 differentiated pre-alpha cells or human islets challenged sequentially with 2.8 or 20 mM glucose with a 60-min incubation for each concentration. Data presented as mean glucagon or insulin secreted per 1000 total cells ± SEM (*n* = 3 biologically independent samples) Significance calculated using a two-tailed paired Student’s *t* test. ESC: embryonic stem cell, DE: definitive endoderm, GTE: gut tube endoderm, PP: pancreatic progenitor, EP: endocrine progenitor, PA: pre-alpha cell, KGF: keratinocyte growth factor, LDN: LDN193189, Alk5i: Alk5 inhibitor II, Repl.: replicating cells.

Our previously published protocol for generating stem cell-derived beta cells (SC-beta cells) produces a small but significant population of pre-alpha cells^8,11^. Figure 1a shows a typical result wherein 27% of the cells are glucose-responsive SC-beta cells and 9% are pre-alpha cells expressing both insulin and glucagon. Kelly et al.^16^ suggested that these pre-alpha cells (referred to as polyhormonal in ref. ^16^) come from progenitors that fail to express NKX6.1. Thus, we sought to modify our beta cell protocol to prevent or reduce induction of NKX6.1 at stage 4. Using the HUES8 embryonic stem cell line, we observed that removal of KGF, SANT-1 and treatment with LDN only on day 2 of stage 3 results in a decrease of NKX6.1-positive cells^11^. In addition, we observed that treatment with LDN on day 1 of stage 4 and incubation with no factors for the remainder of stage 4 resulted in a significant population of Chromogranin A+/NKX6.1− cells. Together these protocol modifications (Fig. 1b) resulted in a large fraction of pre-alpha cells as marked by the coexpression of insulin and glucagon proteins (Fig. 1c). In the HUES8 cell line, this pre-alpha cell optimized protocol produces an average of 62.6 ± 2.3% insulin+ and glucagon+ coexpressing pre-alpha cells and a small percentage (<10%) of monohormonal glucagon-expressing SC-alpha cells (Supplementary Fig. 1). Immunofluorescent staining confirms a high proportion of cells that express both insulin and glucagon protein (Fig. 1d) distributed throughout the cell clusters.

### Pre-alpha cell transcriptional profile

We investigated the transcriptional signature of the pre-alpha populations produced at the end of stage 5 by single-cell RNAseq. Using single-cell sequencing (inDrops)^26^, we profiled 2043 cells from a pre-alpha cell differentiation revealing four distinct cell populations (Fig. 1e). Confirming the immunostaining and flow cytometry analysis, we observed a population of cells that express both insulin and glucagon transcripts, although expression of insulin transcripts was significantly lower than glucagon transcripts (mean tpm of 649 vs. 214,320; Fig. 1f and Supplementary Fig. 2a), indicating that these cells have downregulated insulin expression. This pre-alpha cell population (pink in Fig. 1e) expresses a transcriptional signature more similar to alpha cells than to beta cells (Supplementary Figs. 2b and 3). In addition to expressing insulin and glucagon transcripts, the pre-alpha cells also express transcripts for several markers of alpha cells and lack several key markers for beta cells. For example, pre-alpha cells express transcripts for *ARX*, *IRX1*, and *IRX2*, transcription factors that are expressed in endogenous alpha cells^27^ (Supplementary Fig. 2b), but do not express transcripts for beta cell markers such as *NKX6-1*, *PDX1*, and *PAX4* (Supplementary Fig. 3). Figure 1f shows the relative transcript expression levels of pancreatic hormones in the pre-alpha cell population compared to the major endocrine cell types from human islets.

In addition to the pre-alpha cell population, two minor cell populations are present including a *TPH1-*positive enterochromaffin population^11^ and a nonendocrine population. The presence of these minor populations compared to the large number of pre-alpha cells confirms at the transcriptional level our finding that the majority of cells produced with this protocol are pre-alpha cells. Since the transcriptional profile of pre-alpha cells closely resembles alpha cells, we hypothesized that the pre-alpha cell population may be useful in generating stem-cell-derived alpha (SC-alpha) cells.

### Pre-alpha cells secrete glucagon but not insulin

Due to the transcriptional similarity of pre-alpha cells to primary (cadaveric) alpha cells, we evaluated the functional response of pre-alpha cells to glucose. Pre-alpha cell clusters secrete glucagon under low-glucose (2.8 mM) conditions and suppress glucagon secretion at high glucose (20 mM) concentrations (Fig. 1g). Membrane depolarization induced by high (30 mM) extracellular K^+^ stimulated glucagon secretion in both pre-alpha cells and mature human islet alpha cells. When assessing insulin secretion from these cells with a human specific processed-insulin ELISA, pre-alpha cells do not secrete significant quantities of processed insulin compared to human islets. Although these cells express insulin protein (Fig. 1c), the c-peptide antibody used in flow cytometry and immunofluorescence experiments does not distinguish fully processed insulin from its precursor, indicating that pre-alpha cells do not process proinsulin into insulin.

To analyze the insulin processing in pre-alpha cells, we evaluated proinsulin processing by Western blot (Supplementary Fig. 2c). In human islets, the majority of proinsulin protein is converted to insulin, while in pre-alpha cells, the majority remains as proinsulin. We assessed transcriptional expression of the prohormone converting enzymes PC1 and PC2 (*PCSK1* and *PCSK2* genes) and found that pre-alpha cells expressed *PCSK2* to a much higher degree than they express *PCSK1* (Supplementary Fig. 2b). Thus, pre-alpha cells transcribe the insulin gene and produce proinsulin protein, but do not cleave proinsulin nor secrete mature insulin in significant quantities.

### The pre-alpha cell is a transient state in vitro and in vivo

**Fig. 2:**
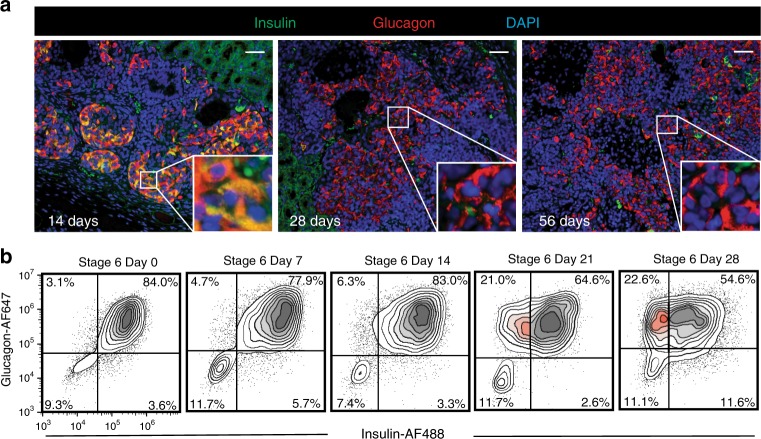
Insulin expression is reduced following transplantation and extended culture in vitro. **a** Expression of insulin and glucagon in grafts after transplantation of pre-alpha cells under the kidney capsule of mice (*n* = 10 animals) at 14 days (left), 28 days (middle) and 56 days (right) post transplant. Inlay shows zoomed-in view of cells at each time point. Coexpression of insulin and glucagon is observed at 14 days. At 28 and 56 days, insulin expression is absent from the majority of cells. Images are representative of *n* = 2 grafts harvested at each time point. Grafts harvested at 4 and 7 days are not shown. Scale bar = 50 μm. **b** Extended culture in vitro results in a fraction of pre-alpha cells reducing insulin expression and continuing to express glucagon (highlighted in red). After 28 days extended culture, about 23% of cells express glucagon but not insulin.

Previous reports demonstrated the presence of a small population of alpha cells in grafts from transplanted SC-beta cell differentiations^8^. We postulated that these alpha cells were derived from the pre-alpha cell side populations present in these SC-beta cell differentiations. As such, we tested the ability of pre-alpha cells generated in our protocol to convert into SC-alpha cells post transplant. We transplanted 5 million pre-alpha cells under the kidney capsule of (*n* = 10) immunocompromised (SCID-beige) mice and observed a reduced expression of insulin protein over the course of 4 weeks. Grafts were retrieved 14, 28, or 56 days after transplantation and assessed for hormone expression using antibodies that react with insulin and glucagon proteins. At 14 days after transplantation, pre-alpha cells continued to express insulin and glucagon proteins (Fig. 2a left, Pearson’s *R* value = 0.57). When grafts were evaluated at 28 days, few insulin protein-expressing cells were observed, whereas glucagon protein-expressing cells persisted (Fig. 2a middle, Pearson’s *R* value = 0.15). This population of monohormonal glucagon-expressing cells were observed for up to 56 days post transplant (Fig. 2a right, Pearson’s *R* value = 0.06). These results suggest that insulin protein expression is reduced in pre-alpha cells and glucagon protein expression is maintained with extended time in vivo. This result is consistent with previous studies which concluded that cells expressing both insulin and glucagon can resolve into alpha cells^20,25,28,29^. To exclude the possibility that the increase in SC-alpha cells observed after transplantation was due to selective replication of an SC-alpha subpopulation and/or concomitant death of pre-alpha cells, we evaluated cell replication and apoptosis during this in vivo maturation (Supplementary Fig. 4). Rarely were TUNEL+/glucagon+ cells observed. Although low levels of Ki67-positive replicating cells were observed, they occurred equally in cells expressing both insulin and glucagon and glucagon-only (Supplementary Fig. 4).

Given the ability of pre-alpha cells to reduce insulin protein expression after transplantation, we tested whether this conversion can occur in vitro. After generation of pre-alpha cells, we continued culturing the cells for an additional 4 weeks in media without growth factors. During this extended culture period, cells were sampled at 7-day intervals and assessed for insulin and glucagon protein expression by flow cytometry. As shown in Fig. 2b, the vast majority of these cells (>80%) expressed both insulin and glucagon proteins at the beginning of the culture period. After 14 days, little change had occurred as >80% of the cells still expressed insulin and glucagon protein. After 21 days, a population of insulin-, glucagon+ cells appears with ~20% of the cells expressing only glucagon protein. This population of monohormonal SC-alpha cells that appeared at day 21, persists at day 28 of extended culture. These results indicate that upon extended culture in vitro, a fraction of pre-alpha cells reduce insulin protein expression and become SC-alpha cells.

### PKC activation promotes maturation of SC-alpha cells

**Fig. 3:**
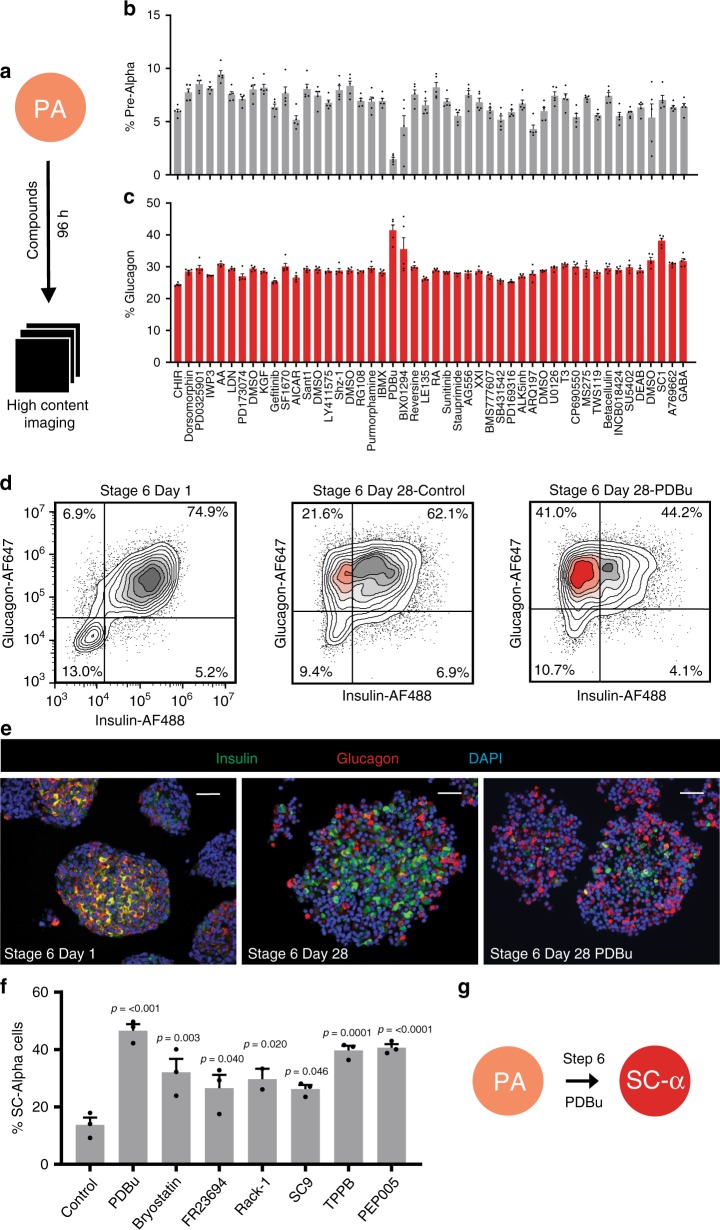
Screen to identify compounds that promote alpha cell identity. Small molecules targeting known pathways (43 compounds) were incubated with pre-alpha cells in quintuplicate for 96 h. **a** Schematic of screening approach. Primary screening results showing **b** the percentage of cells expressing both insulin and glucagon and **c** the percentage of cells expressing only glucagon. The data are presented as mean ± SEM (*n* = 5 biologically independent samples). The PKC activator PDBu significantly reduced the percentage of pre-alpha cells and increased the percentage of cells expressing glucagon. **d** Representative flow cytometry results of pre-alpha cells at stage 6 day 1, and stage 6 day 28 control (no treatment), or 28 days treatment with the PKC activator PDBu. **e** Immunofluorescence of clusters treated with and without PDBu for 28 days. Scale bar = 50 μm. **f** Additional PKC activators evaluated for their effect on pre-alpha cells (stage 6 day 1). All PKC activators increased the percentage of SC-alpha cells. The data are presented as mean ± SEM, significance calculated using an ordinary one-way ANOVA with Dunnett multiple comparison test (*n* = 3 biologically independent samples). **g** Stage 6 of protocol converts pre-alpha cells into SC-alpha cells.

Given that pre-alpha cells can convert to SC-alpha cells in vivo and in vitro, we sought to identify signals that promote this conversion by performing a small-molecule screen. HUES8 embryonic stem cells were differentiated using the pre-alpha cell optimized protocol to the end of stage 5 (stage 6 day 1), arrayed into 384-well plates and incubated with a custom 43-member small-molecule library (Supplementary Table 3) where compounds were chosen for their activity in targeting signaling pathways. After 96 h of treatment (stage 6 day 5), cells were fixed and stained for insulin and glucagon (Fig. 3a). High-content imaging was used to quantify the dispersed (2D) cell populations for the percentage of cells expressing each hormone individually and the percentage of pre-alpha cells marked by expression of both hormones (Fig. 3b, c). The effect of each compound was evaluated in quintuplicate assays. The protein kinase c (PKC) activator phorbol 12,13-dibutyrate (PDBu) decreased the percentage of insulin protein-expressing cells and increased the percentage of glucagon-expressing cells compared to vehicle controls. To confirm that the effects of this compound were not unique to the planar assay format, PDBu was evaluated in the final stage of the 3D directed differentiation protocol. Over the course of a 28-day treatment, PDBu induced a significant population of pre-alpha cell clusters to reduce insulin protein expression compared to control (33 ± 6% vs. 16 ± 5%; *n* = 5; Fig. 3d and Supplementary Fig. 5). The resulting cell population contained significantly more alpha cells that did not coexpress the insulin protein (Fig. 3e).

To further explore the specificity of PKC activation, a small library of known PKC activators was evaluated. This structurally diverse set of compounds have all been reported to have PKC activating abilities. We added pre-alpha cell clusters (stage 6 day 1) to six-well plates and treated with either vehicle or each PKC activator for 28 days and assessed the percentage of monohormonal, glucagon-expressing alpha cells (Fig. 3f and Supplementary Fig. 6). While the control condition resulted in only 13.7% SC-alpha cells, treatment with each PKC activator resulted in a significant increase in the percentage of SC-alpha cells, though none more so than PDBu. To evaluate the stability of the shift from pre-alpha cells to SC-alpha cells, we withdrew PDBu from cells for 7 days and evaluated the percentage of monohormonal glucagon-expressing SC-alpha cells (Supplementary Fig. 7). Withdrawal of PDBu did not significantly affect the percentage of SC-alpha cells in our population. These results demonstrate that activation of PKC stably reduces the expression of insulin in the INS+/GCG+ population thereby accelerating the conversion process of pre-alpha cells to SC-alpha cells in vitro (Fig. 3g).

As previously described, differentiation of hPSCs to beta cells results in a side population of pre-alpha cells. To this end, we evaluated the effect of PKC activation on the differentiation of SC-beta cells. Treatment of stage 6 SC-beta cells with the PKC activator PDBu did not have a significant effect on the number of beta cells in the population (Supplementary Fig. 8a). However, when treated with PDBu, the pre-alpha side population in the differentiation was reduced and the percentage of SC-alpha cells was increased (Supplementary Fig. 8b). Because previous reports pointed to variability when using different cell lines^30^, the robustness of this SC-alpha protocol was evaluated in the 1016 iPS cell line. Differentiated 1016 cells generate a similar percentage of SC-alpha cells (Supplementary Fig. 9) showing that the protocol is able to direct another stem cell line to SC-alpha cells using this protocol. To establish the performance of our SC-alpha cell protocol in comparison to previously published reports, we performed a head-to-head comparison where SC-alpha cells were generated with our protocol or the Rezania protocol^15^ in order to assess protocol efficiency and the functional performance of the resulting cell populations (Supplementary Figs. 1, 10, 11, 12, 13). Using the HUES8 cell line, the percentage of SC-alpha cells produced was improved more than eightfold over previously reported protocols and demonstrates improved functional responses to glucose.

### Molecular characterization of pre-alpha and SC-alpha cells

**Fig. 4:**
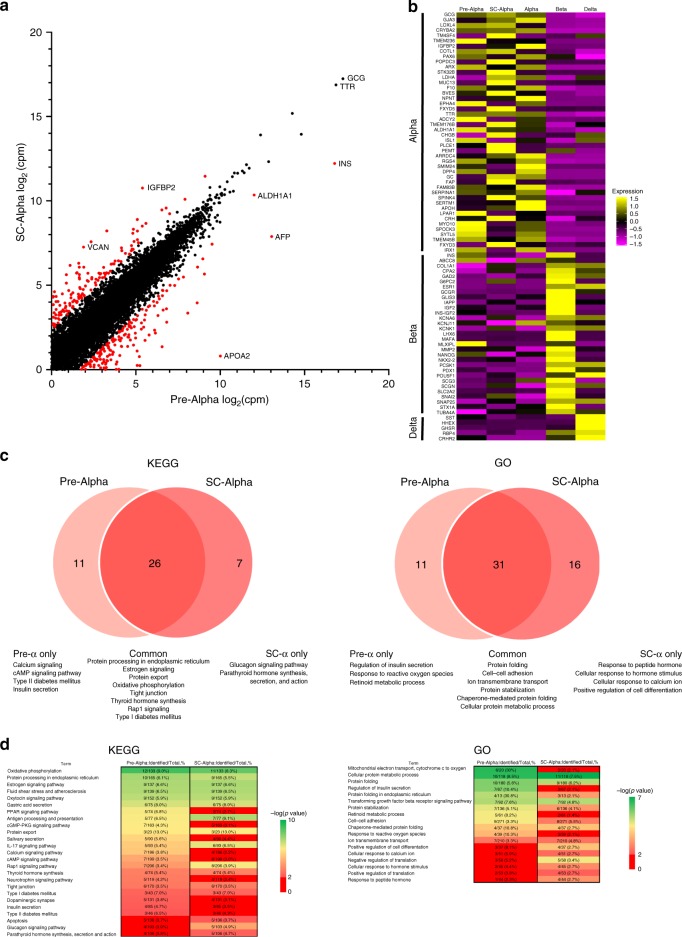
Molecular characterization of pre-alpha cells and SC-alpha cells. **a** Scatterplot showing relative expression of pre-alpha (*x*-axis) and SC-Alpha (*y*-axis) transcript counts. Both axes are log2(cpm). Genes with fold change greater than 0.5 or less than −0.5, and *p* value < 0.01 as calculated using a Wilcoxon rank sum test, are highlighted in red. **b** Heatmap showing pre-alpha and SC-Alpha cells in comparison with human alpha, beta, and delta cells. Top 46 genes are alpha cell specific, middle 31 genes are beta cell specific, and bottom 5 genes are delta cell specific. Coloring is based on *z*-score from 1.5 as high (yellow) to −1.5 as low (purple). **c** Venn diagrams comparing KEGG pathway (left) and Gene Ontology-Biological Process (right) database overlap between the highest expressing 150 genes in pre-alpha and SC-Alpha cells. Significant terms defined as *p* value < 0.01 as calculated using an EASE Score (modified Fisher Exact test). **d** Heatmaps showing selected pathways from KEGG and Gene Ontology-Biological Process terms for pre-alpha and SC-Alpha cells. Columns show number of genes mapped to the pathway divided by number of possible genes in the pathway, in addition to its percentage. Scale is from –log(*p* value) of 2 (light red) to 10 (green) for KEGG and –log(*p* value) of 2 (light red) to 7 (green) for GO-BP. All pathways that were not significant as calculated using an EASE Score (modified Fisher Exact test), i.e. –log(*p* value) from 0 to 2, are labeled in red. Full heatmaps are shown in Supplementary Figs. 14 and 15.

To investigate the transcriptional changes that occur as cells transition from pre-alpha cells to SC-alpha cells, we performed single-cell RNAseq on pre-alpha cells and SC-alpha cells (Fig. 4). On a global level, the transcriptional profile of pre-alpha cells and SC-alpha cells are remarkably similar with insulin expression being a notable exception (Fig. 4a). Analysis of 82 islet specific genes in comparison to human islet expression patterns further confirms the similarity of both pre-alpha cells and SC-alpha cells to primary human alpha cells (Fig. 4b). These results suggest that the conversion of pre-alpha cells to SC-alpha cells represents a subtle maturation process rather than a cell fate change. Consistent with our global gene expression analysis, a KEGG and gene ontology pathway analysis demonstrates significant similarity between pre-alpha and SC-alpha cells (Fig. 4c, d and Supplementary Figs. 14 and 15) with the most significant pathways common between both cell types. These top pathways are consistent with the function of alpha cells and include protein export, protein processing in the ER, protein folding, and cellular signaling pathways. Despite these similarities, some differences were observed. Pre-alpha cells uniquely express genes enriched in the insulin secretion and metabolic stress pathways, while SC-alpha cells uniquely express genes enriched in the glucagon secretion and hormone action pathways.

Using pseudotime analysis, we established a sequence of transcriptional changes that occur as cells progress from pre-alpha to SC-alpha cells (Supplementary Fig. 16). The transition from pre-alpha to SC-alpha results in the sequential decrease of stress-related genes *VTN* and *AFP* followed by beta and delta cell genes *INS*, *TSPAN1*, *GAL*. Finally, alpha-cell-associated genes *PLIN2*, *TXNIP*, and *GC* are upregulated. To further examine the role of PKC activation on these transcriptional changes, we performed an additional scRNA-seq experiment to compare the transcriptional profile of SC-alpha cells generated with PDBu to untreated SC-alpha cells which resulted from a spontaneous conversion from pre-alpha to SC-alpha cells. (Supplementary Fig. 17a). The tSNE plots from stage 6 day 28 cells generated with and without PDBu resulted in three major overlapping cell populations indicating significant similarity in their transcriptional profile. The predominant population expresses transcripts that are known markers of cadaveric alpha cells (*ARX*, *IRX1*, *IRX2*)^27^ (Supplementary Fig. 17b). Very few genes are differentially expressed between the SC-alpha generated with and without PDBu (Supplementary Fig. 17c and Supplementary Table 2). Of the genes that are differentially expressed, insulin transcript is downregulated 2.3-fold in cells treated with PDBu. Glucagon transcripts were not significantly affected by PDBu treatment.

To evaluate the protein expression levels of insulin and glucagon, we performed Western blots on cell lysates from cells treated with PDBu (Supplementary Fig. 18). Cells treated with PDBu had a higher expression of glucagon protein compared to untreated cells. In untreated samples, glucagon expression is decreased. As we observed in our flow cytometry analysis (Fig. 3d), the Western blot confirms that PDBu treatment decreases the level of insulin protein expression (Supplementary Fig. 18).

### Structural and functional characterization of SC-alpha cells

**Fig. 5:**
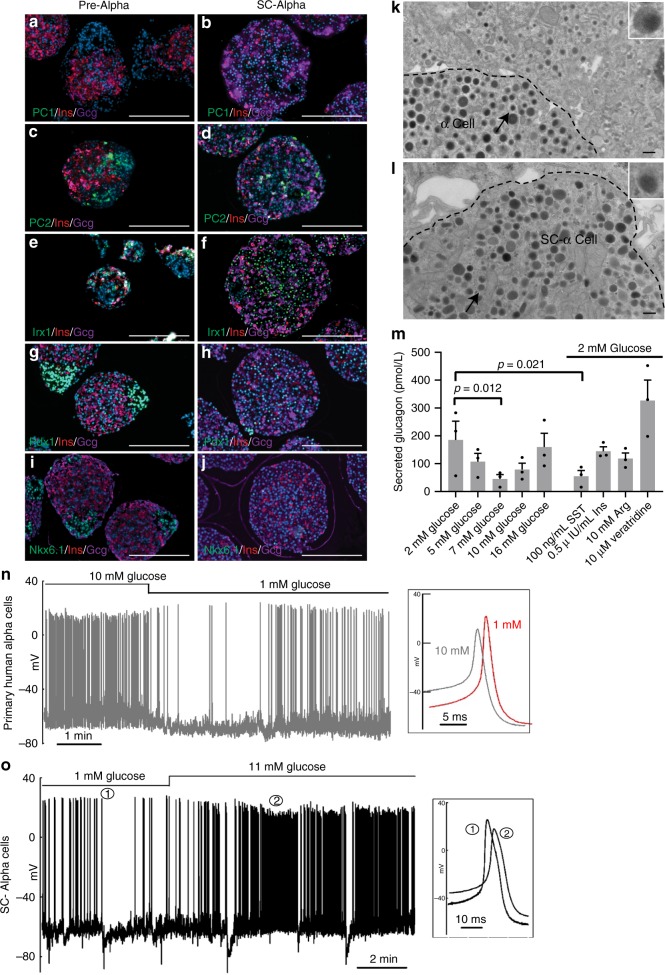
Characterization of SC-alpha cells. Pre-alpha (**a**, **c**, **e**, **g** and **i**) and SC-alpha cells (**b**, **d**, **f**, **h**, and **j**) stained for PC1 (**a** and **b**), PC2 (**c** and **d**), IRX1 (**e** and **f**), PDX1 (**g** and **h**), and NKX6.1 (**i** and **j**). Scale bar for **a**−**j** = 200 μm. The ultrastructure and granule morphology of human cadaveric alpha cells (**k**) and SC-alpha cells (**l**) as assessed by electron microscopy are similar. Arrows indicate granules; dashed line denotes the cell boundary. Inlay shows granule morphology. Scale bar for **k** and **l** = 500 nm. **m** SC-alpha cells secrete glucagon in response to glucose and are inhibited by somatostatin (SST). SC-alpha cell response to the glucagon secretagogue veratridine. Data are presented as mean ± SEM, significance calculated using a paired ratio Student’s *t* test (*n* = 3 biologically independent samples). **n** Representative electrophysiology recording of primary human alpha cell showing electrical activity in response to low (1 mM) and high (10 mM) glucose. Amplitude differences at low and high glucose are shown in the inlay (representative; *n* = 5 biologically independent cells). **o** Representative electrophysiology recording of SC-alpha cell showing electrical activity in response to low (1 mM) and high (11 mM) glucose. Amplitude differences at low and high glucose are shown in the inlay (representative; *n* = 30 biologically independent cells).

SC-alpha cells express known markers of alpha cells including GCG, PDX1, IRX, PC2, but do not express beta cell-specific markers PC1 or NKX6-1 (Fig. 5a−j). Electron microscopy reveals that the ultrastructure of SC-alpha cells resembles that of human primary alpha cells. In human islets, glucagon granules in alpha cells are dark and diffuse with round cores with an average size of 242 ± 8 nm (Fig. 5k), which is smaller than the condensed insulin granules of the beta cell^31^. In SC-alpha cells, the secretory granules have a similar ultrastructure to alpha cells with an average size of 214 ± 12 nm (Fig. 5l).

To evaluate the ability of SC-alpha cells to respond to physiological conditions, we screened a number of glucagon secretion modulators (Fig. 5m). Like mature human alpha cells^32–36^, glucagon secretion in SC-alpha cells is inhibited by glucose, an effect that is maximal at 7 mM glucose with higher glucose concentrations being less inhibitory (Fig. 5m). This pattern of glucagon secretion in response to increasing glucose concentrations has been reported by others^37^. Glucagon secretion from SC-alpha cells is suppressed in the presence of exogenous somatostatin, normally released within the islets by the delta cells, unaffected by exogenous insulin and arginine and stimulated by veratridine, an activator of voltage-gated Na^+^ channels. Arginine evokes a transient stimulation of glucagon secretion ex vivo in mature alpha cells and time-resolved measurements may therefore be necessary to detect its stimulatory effect^38^.

We also sought to evaluate the stimulatory capacity of SC-alpha cells using electrophysiology. Using a fluorescent reporter cell line that expresses mCherry under the endogenous glucagon promoter, we identified alpha cells for patch clamp experiments and compared the electrical activity of these cells to primary human alpha cells in response to changing glucose concentrations. Like primary alpha cells, SC-alpha cells generate spontaneous overshooting action potentials in the presence of 1 mM glucose (Fig. 5n, o). In the SC-alpha cells, these action potentials reflect activation of voltage-gated Na^+^, Ca^2+^, and K^+^ channels, similar to what has been demonstrated in adult human alpha cells^37^. The effects of high glucose (10−11 mM) were also similar: slight depolarization of the interspike membrane potential, an increase in action potential frequency and a reduction in action potential height (Fig. 5n, o inlays). These effects of high glucose are similar to those reported in mouse alpha cells and indicate that glucagon secretion may be influenced by action potential firing in a amplitude- rather than frequency-dependent fashion^39^. In all, the results demonstrate that SC-alpha cells resemble human primary alpha cells in their transcriptional profile, glucagon granule morphology, electrophysiology and physiological response to several (but not all) glucagon secretion modulators in static incubations.

### SC-alpha cells reduce hypoglycemia in transplantation models

**Fig. 6:**
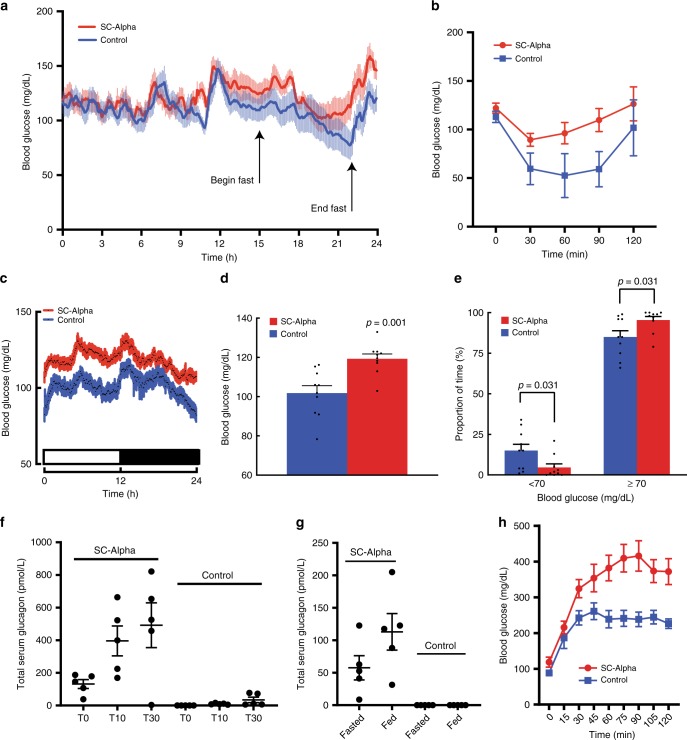
In vivo characterization of SC-alpha cell transplantation. **a** Fasting-induced hypoglycemia in control (*n* = 9 animals) and SC-alpha transplanted mice (*n* = 9 animals). Continuous glucose monitoring for 24-h period at 5 weeks post transplant. Food was removed after 15 h and restored at 22 h. SC-alpha-cell-transplanted mice are protected from hypoglycemia. **b** Insulin tolerance test on control (*n* = 9 animals) and SC-alpha (*n* = 9 animals) transplanted mice at 6 weeks post transplant. SC-alpha-cell-transplanted mice are protected from hypoglycema. **c** Continuous glucose monitoring of mice for 4 weeks reveals that mice transplanted with SC-alpha cells (*n* = 10 animals) have elevated blood glucose compared to control animals (sham surgery, *n* = 10 animals). Graph represents the average daily blood glucose value at 5-min intervals for all animals in each treatment group. Each data point represents the average of 280 CGM readings (10 mice × 28 days). **d** In transplanted mice, SC-alpha cells reduce instances of hypoglycemia in mice. Average blood glucose reading by CGM over 28-day period (*n* = 10 animals for each group). **e** Percentage of time over 4 weeks spent in range (≥70 mg/dL) and hypoglycemic (<70 mg/dL) (*n* = 10 animals for each group). **f** Total serum glucagon levels in response to an arginine bolus at 4 weeks post transplant. SC-alpha-transplanted mice (*n* = 5 animals) and control animals (*n* = 5 animals) were fed ad libitum and injected i.p. with arginine. Total serum glucagon levels were measured at time 0 (T0), 10 and 30 min post injection. **g** Fasting and fed total serum glucagon levels for control (*n* = 5 animals) and SC-alpha (*n* = 5 animals) transplanted animals. Fasted mice were deprived of food for 6 h before blood collection. Blood was collected from fed animals 2 h after food was restored. **h** Glucose tolerance test in control mice (*n* = 5 animals) and SC-alpha-transplanted mice (*n* = 5 animals). All data are presented as mean ± SEM. Significance was calculated using a two-tailed unpaired Student’s *t* test.

We evaluated the potential utility of these cells to modulate physiology in vivo by transplanting SC-alpha cells. Using a continuous glucose monitor (CGM), we evaluated the interstitial glucose concentrations in animals at 5-min intervals during a period of fasting-induced hypoglycemia. Control animals (sham surgery) exhibited hypoglycemia after 6 h of fasting while mice transplanted with SC-alpha cells were protected from hypoglycemia (Fig. 6a). In SC-alpha-transplanted animals, blood glucose levels begin to return to baseline before food was restored while control animals continued to decrease blood glucose until food was restored. Furthermore, when administered an i.p. bolus of exogenous insulin, SC-alpha-transplanted animals are protected from hypoglycemia (Fig. 6b). To evaluate the effect of transplanted SC-alpha cells on long-term blood glucose values, we measured interstitial blood glucose continuously starting 4 weeks after transplantation and continuing until week 8. During this time, mice were maintained under normal housing conditions with 24-h light/dark cycles and feeding ad libitum. At the end of the 4-week observation period, CGM glucose readings for all mice in a cohort were averaged and represented as an average blood glucose value per 5-min interval throughout a standard 24-h period with the associated standard error (Fig. 6c). As has previously been reported^40–43^, control mice exhibited a characteristic fluctuation pattern in blood glucose values over a standard 24-h period with elevated blood glucose levels during active/feeding periods (dark) and lower blood glucose levels during resting periods (light). In SC-alpha-transplanted mice, we observed a similar pattern of glucose fluctuations, although the average blood glucose concentrations were elevated compared to control mice (Fig. 6d). Transplantation of SC-alpha cells does not perturb the normal circadian regulation of blood glucose concentrations in these animals but the presence of additional glucagon secreting cells raises the basal blood glucose concentrations in these animals. Further evaluation of circadian and ultradian patterns in blood glucose levels confirms that there is no significant shift in periodicity of these previously reported glucose rhythms (Supplementary Fig. 19).

The sum result of SC-alpha cell transplantation is an elevation of blood glucose levels and a reduction in the proportion of time that mice spend with blood glucose levels in the hypoglycemic range (<70 mg/dl). As shown in Fig. 6e, mice that have received SC-alpha cell transplants spend on average 5 ± 2% of the time in hypoglycemia as compared to 15 ± 4% for control animals. These results suggest that transplantation of SC-alpha cells may have the ability to offer some protection from hypoglycemia.

To further demonstrate that the protective effect of SC-alpha cells was due to glucagon signaling, we evaluated the ability of SC-alpha cells to secrete glucagon in vivo in response to arginine stimulation and in response to a mixed meal. Control mice and mice transplanted with SC-alpha cells were injected i.p. with an arginine solution to stimulate glucagon secretion from SC-alpha cells. After arginine injection, serum glucagon levels raised more than twofold (Fig. 6f and Supplementary Fig. 20). In comparison to control animals, both the basal and stimulated glucagon levels in these animals are several fold higher in the SC-alpha-transplanted animals suggesting that the majority of glucagon observed in these studies was secreted from the transplanted SC-alpha cells. As has been previously reported for alpha cells^44,45^, SC-alpha cells secrete glucagon in response to administration of a mixed meal. After refeeding, SC-alpha transplanted mice demonstrated a greater than twofold increase in serum glucagon (Fig. 6g). Given the significantly higher circulating glucagon observed in SC-alpha-transplanted animals under both fasting and fed conditions, we sought to evaluate the influence of transplanted SC-alpha cells on glucose tolerance and clearance. In a glucose tolerance test (GTT), SC-alpha-cell-transplanted mice demonstrated elevated fasting blood glucose and peak glucose levels, with impaired glucose clearance (Fig. 6h). Given that circulating glucagon levels in these animals (basal and stimulated) are several fold higher than control animals (Fig. 6f, g), it is not surprising that blood glucose levels are elevated during glucose challenge. Taken together these results demonstrate the function of SC-alpha cells upon transplantation and the ability of these cells to modulate blood glucose metabolism and physiology.

## Discussion

The promise of regenerative medicine lies in the ability to produce tissues to replace damaged or diseased tissues. Diabetes has been a leading candidate for this approach because a single-cell type, the beta cell, plays such a critical role in the disease. This led to a focus on the generation of pancreatic beta cells and although much progress has been made, there are potential advantages in adding other cell types to treat diabetes.

Numerous studies highlight the importance of multiple islet cell types in regulating insulin secretion^46,47^. Paracrine signaling from alpha cells establishes a set point for insulin secretion^48^, and glucagon is important for beta cell regeneration in a zebrafish model^49^. Recent studies suggest that alpha cell dysfunction plays a role in chronic hyperglycemia in patients with T1D due to hyperglucagonemia^4^. Additionally, a defective alpha cell response to hypoglycemia is part of the impaired counter-regulatory response to hypoglycemia observed in T1D ^7^. After cessation of insulin secretion in nondiabetics, glucagon is the first line of defense against hypoglycemia prior to activation of sympathetic responses. However, this response is altered in T1D due to alpha cell dysfunction, resulting in increased episodes of hypoglycemia. In T1D, insulin concentrations are dependent on insulin dosing while the sympathetic counter-regulatory response is impaired by prior hypoglycemia^50^. The ability of SC-alpha cells to protect against hypoglycemia and increase circulating glucagon levels in response to physiological cues may represent an avenue for therapeutic intervention in hypoglycemia. Although we have not evaluated the long-term impacts of SC-alpha cell transplantation in these studies, the high levels of circulating glucagon observed in response to SC-alpha cell transplantation are likely to result in compensatory glucagon resistance^15^.

Despite mounting evidence that defects in beta cell function do not fully account for the dysregulation observed in diabetes^4,7^, current therapies for diabetes (both type 1 and type 2) have focused almost exclusively on the insulin-producing beta cell. For example, insulin replacement therapy used in patients with type 1 and type 2 diabetes seeks to replace insulin that would normally be produced by beta cells. Few therapies are currently on the market that target other cell types in the pancreas. GLP-1 receptor agonists such as exenatide have been shown to reduce glucagon secretion from the pancreas, and it has been suggested that exenatide acts directly through alpha cells^51^. Furthermore, the mechanism by which glucose regulates glucagon secretion remains unclear and has been attributed to both intrinsic and paracrine effects. The ability to generate SC-alpha cells enables the exploration of these mechanisms in the absence of paracrine interactions from beta and delta cells.

Reduced glucagon levels have been observed following treatment of type 2 diabetic patients with DPP-4 inhibitors, and since DPP-4 inhibition results in increased circulating endogenous GLP-1, a similar mechanism for GLP-1 receptor agonists has been suggested^52^. Furthermore, there have been multiple attempts to produce glucagon receptor antagonists, since the high glucagon levels observed in type 2 diabetics may contribute to hyperglycemia. On the other hand, glucagon secretagogues, such as the TRG5 agonist ABT-777, could potentially be used in the treatment of hypoglycemia for patients with type 1 diabetes. Despite these advances, much remains unknown regarding the druggability of alpha cells and no good models for functional human alpha cells exist for in vitro studies. Future advances could be accelerated by the availability of renewable sources of the alpha cells reported here.

In this study, we focus on the generation of pancreatic alpha cells as a step toward making all islet cell types, development of cell-based therapies that prevent hypoglycemia and better regulate plasma glucose concentrations, and co-culture experiments of alpha and beta cells to better understand the interplay between these cell types in islet function and disease. The scalable 3D protocol described here has some advantages over previous reports^15^, including the absence of exogenous growth factor matrices such as Matrigel™. Moreover, this protocol enables the production of hundreds of millions of SC alpha cells (~1 SC-alpha cell for every three cells seeded), which is much more efficient that 2D cultures reported.

In producing SC-alpha cells in vitro, we describe the generation of pre-alpha cells that express both insulin and glucagon proteins. Similar polyhormonal cells have been described in a number of different situations, including normal development^25^, in pancreatitis and under stress conditions^23^, and as an unexpected side population in directed differentiation protocols^8,11^. Uncertainty regarding the nature of these cells has existed with some reports describing these cells as immature beta cells, others describing these cells as intermediates for alpha cells and still others describing these cells as a hybrid cell type with features of both alpha and beta cells. Cells expressing both insulin and glucagon have been observed early in mouse, rat, and human development during the primary transition of endocrine differentiation in the pancreas^28,53,54^. These early instances of polyhormonal cells do not persist into adulthood and have been postulated to resolve into alpha cells^25^. Furthermore, in humans, the existence of polyhormonal cells in the healthy adult pancreas has not been reported, although there are reports of polyhormonal cells in human pancreatic tissues associated with stress and disease^23,24^. We propose a nomenclature for alpha-like cell populations allowing the term “alpha-like” to describe any cell that has a transcriptional signature resembling an alpha cell (*GCG*+/*ARX*+/*IRX1*+) and designating two subsets of alpha-like cells: “pre-alpha” cells and “SC-alpha cells” defined by their expression or lack of INS. While alternate mechanisms for generating human pancreatic alpha cells may not require the generation of this pre-alpha intermediate, the present study demonstrates the potential of pancreatic pre-alpha cells to produce functional alpha cells.

As shown here, activation of PKC resulted in the maturation of pre-alpha cells into glucagon-expressing SC-alpha cells. PKC activation has been reported to enhance glucagon secretion from mouse and human islets through calcium-dependent exocytosis^51,55^. Further, a role for PKC in the transcription and protein expression of glucagon has been reported^56^. Our studies further establish the importance of PKC in alpha cells and establishes a role for PKC activation in alpha cell identity and maturation. The transcriptional changes occurring as cells mature from pre-alpha cells to SC-alpha cells contain several genes implicated in PKC signaling. PKC activation plays a role in cell stress response^57^, tetraspanin regulation^58^, and a role in Vitamin D and calcium regulation^59^. Our studies identifying PKC activation as an important signaling pathway in alpha cell identity and maturation is limited by the lack of published studies linking PKC signaling to alpha cell function. Further studies are needed to identify the precise mechanistic action of PKC activation in alpha cell maturation. These studies, as well as studies combining SC-alpha and SC-beta cells, will now be possible.

## Methods

### Cell culture

Human pluripotent stem cells (HUES8 and 1016 cell lines) were maintained as previously described^8^. Briefly, cells were adapted to a highly scalable 3D culture system using spinner flasks and maintained using mTeSR media. Suspension cultures were established by seeding 150 million cells in mTeSR media with 10 μM Y27632 and maintained at 70 rpm in a humidified incubator at 37 °C and 5% CO_2_. Media was changed at 48 h to mTeSR without Y27632. Cells were passaged every 72 h by dispersing to single cells using Accutase and seeded into fresh mTeSR with Y27632. The Harvard University Embryonic Stem Cell Research Oversight Committee and the Mayo Clinic Stem Cell Research Oversight Committee reviewed and approved all work involving human pluripotent stem cells carried out in this manuscript including review of compliance with informed consent.

### Directed differentiation

Differentiations were initiated 72 h after seeding into mTeSR at a density of 0.5 million cells per mL. Clusters were allowed to settle at the bottom of the spinner flask and the media was removed by aspiration. Protocol-specific media was introduced to the spinner flask with appropriate growth factors and the flask was returned to the incubator with stirring. Cells were directed sequentially to a definitive endoderm (DE), gut tube endoderm (GTE), pancreatic progenitor (PP), endocrine progenitor (EP) and pre-alpha (PA) population as described below. Liquid media (S1, S2, S3) composition is as previously reported^8^. Media changes are as follows—Day 1: S1 + 100 ng/mL Activin A + 3 μM CHIR99021. Day 2: S1 + 100 ng/mL Activin A. Day 4: S2 + 50 ng/mL KGF. Day 6: S3 + 2 μM RA. Day 7: S3 + 2 μM RA + 200 nM LDN193189. Day 8: S3 + 200 nM LDN193189. Days 9, 11: S3 (no additional factors). Days 13, 15, 17, 19: S3 + 10 μM Alk5i. Days 20−48: S3 + 500 nM PDBu (feed on even days only). Modifications of LDN concentrations were found to enhance differentiation percentages in the 1016 cell line. LDN concentrations of 1 µM were used at days 6, 7, and 8. Additional information regarding growth factors and small molecules can be found in Supplementary Table 1.

### Flow cytometry

Cells were collected for flow cytometry analysis at various time points throughout the differentiation process. Cells were dispersed with TrypLE Express at 37 °C for 15 min after which TrypLE was quenched with S3 media. Cells were fixed in 4% paraformaldehyde (PFA) for 20 min and subsequently stored in PBS at 4 °C until staining. Staining was performed by permeabilizing the cells in block solution (PBS + 0.1% Triton X-100 + 5% donkey serum) for 40 min. Cells were then incubated with primary antibodies in block solution for 1 h at room temperature (RT), washed twice with PBST and incubated with secondary antibody in block solution for 1 h at RT. Cells were then washed three times and resuspended in PBST at a concentration of 1 × 10^6^ cells/mL. Stained cells were analyzed using the Accuri C6 or Attune flow cytometers. FlowJo v10 and Attune NxT ver 2.6 software were used to process the respective flow cytometry data. Insulin was assessed using an antibody that recognizes the c-peptide epitope from the Iowa Hybridoma Bank at a concentration of 1:300 (DSHB, GN-ID4). Glucagon was assessed using an antibody that recognizes the GLP-2 (Santa Cruz, SC-7781) or glucagon (Sigma, G2654) epitopes, and used at a concentration of 1:300 or 1:1000, respectively. The antibodies used recognize epitopes for both proinsulin and processed insulin as well as proglucagon and processed glucagon. For simplicity, the nomenclature insulin and glucagon is used throughout the text. Secondary antibodies used were donkey-anti-rat-Alexa Fluor 488 (Invitrogen A-21208, 1:500) and either donkey-anti-goat-Alexa Fluor 647 for GLP-2 (Invitrogen A-21447, 1:500) or donkey-anti-mouse-Alexa Fluor 647 for glucagon (Invitrogen A-31571, 1:500). The gating strategy for flow plots is shown in Supplementary Fig. 21.

### Immunofluorescence

Cell clusters or kidney grafts were fixed in 4% PFA for 1 h, washed and embedded in Histogel (clusters) and subsequently embedded in paraffin and sectioned. Sections were stained for insulin and glucagon using the antibodies described above and mounted using Fluoromount-G with DAPI (4′,6-diamidino-2-phenlindole). Clusters were imaged using an EVOS FL Auto 2 imaging station.

### Western blot

Stage 6 clusters at different time points and human islets were collected into cell lysis buffer (10 mM Tris-HCl [pH 8.0], 10 mM NaCl, and 0.5% NP-40) containing protease inhibitor (Roche) for 30 min on ice. Lysates were centrifuged at 12,000 × *g* for 20 min at 4 °C, the resulting supernatants and RH Insulin protein (ALPCO) were separated by electrophoresis and Western blotting using antibodies against insulin (Cell Signaling Technology 8138, 1:1000), Glucagon (Abcam ab82270, 1:1000), and GAPDH (Cell Signaling Technology 5174S, 1:5000). A protein ladder (Bio-Rad 1610377) was run to determine band sizes.

### Single-cell RNA sequencing

Single-cell mRNA sequencing was carried out using the inDrops platform^26,60^ on 5000 cells, as previously described using “v3” beads (1-cell bio), or the 10X Genomics Chromium system using the 3′ v3 library kit on >2000 cells per sample. Samples were collected at stage 6 day 0 and stage 6 day 28 from both PDBu+ and PDBu− conditions. Libraries were prepared from each sample and sequenced on a NextSeq 500 or HiSeq 4000. Sequencing was processed using the inDrops pipeline (https://github.com/indrops/indrops/) to generate UMIFM read counts or Cell Ranger v.3.0.2 (https://software.10xgenomics.com/single-cell/overview/welcome) to perform barcode processing and single-cell gene UMI counting.

For inDrops: Read counts were converted to transcript per million (tpm) values, excluding genes that contribute more than 2% of reads in any given cell. High-variance genes were identified as previously described^26^ and used for principal component analysis (PCA). tSNE projection was carried out using the first 25 components (not rescaled to unit variance), using the Python wrapper of the Barnes-Hut C tSNE implementation (https://github.com/lvdmaaten/bhtsne). Clustering was performed using the principal components as input for diffusion map embedding and Louvain community detection, as implemented in Scanpy^61^. For 10X Chromium: Data were analyzed using Seurat v.3^62,63^. Briefly, cells were removed if they had <500 detected genes or contained >25% mitochondrial content. The remaining cells were regressed for variance in mitochondrial genes. Raw UMI counts were normalized per million total counts and log-transformed, with additional batch correction performed using the Seurat functions FindIntegrationAnchors and IntegrateData. A tSNE projection was carried out using the first 20 components, defining clusters which correlated with pre-alpha (*GCG*+, *INS*+) cells and alpha (*GCG*+, *INS*−) cells. Only these clusters were input into Monocle 2 for further analysis. Trajectory analysis was performed in Monocle 2 using semi-supervised ordering, identifying 50 genes that covaried with a change in insulin expression^64^. These genes were used to order the pre-alpha and alpha cells along a pseudotime trajectory. Hierarchical clustering was performed using the hclust function in R, which uses an agglomerative approach to group using the default complete linkage method. Venn diagrams and heatmaps were created by performing gene ontology analysis using David 6.8 for Gene Ontology-Biological Process terms and ConsensusPathDB-human release 34 for KEGG (Kyoto Encyclopedia of Genes and Genomes) pathway analysis. Figures were created in Microsoft Excel 2010.

### Hormone secretion assays

Human islets (Prodo Laboratories) or differentiated pre-alpha or SC-alpha cells were washed twice in low-glucose (2.8 mM) Krebs Ringer (KRB) buffer (128 mM NaCl, 5 mM KCl, 2.7 mM CaCl_2_, 1.2 mM MgSO_4_, 1 mM Na_2_HPO_4_, 1.2 mM KH_2_PO_4_, 5 mM NaHCO_3_, 10 mM HEPES (4-(2-hydroxyethyl)-1-piperazineethanesufonic acid), 0.1% BSA (bovine serum albumin) in MilliQ). Cells were then loaded into 24-well transwell inserts and fasted in low-glucose KRB for 1 h at 37 °C. Clusters were washed once in low-glucose KRB and then incubated in low-glucose KRB for 1 h at 37 °C. After incubation, the supernatant was collected and stored at −20 °C until analysis. Cells were then transferred to high glucose KRB (20 mM) for 1 h at 37 °C and the supernatant was collected and stored. Cells were then transferred to low-glucose KRB with 30 mM KCl to observe depolarization conditions. Cells were incubated in this buffer for 1 h and the supernatant was collected. Cells were then dispersed and counted using a Vi-CELL automated cell counter. Collected supernatants were analyzed by ELISA for human insulin (ALPCO) and/or total glucagon (Mercodia) concentrations and normalized for cell number. For animal transplant studies, total serum glucagon is measured since mouse and human glucagon sequences are identical.

### Chemical screen

Pre-alpha cell clusters were dispersed using TrypLE Express for 15 min and quenched using S3 media. The resulting single-cell suspension was arrayed into a 384-well plate, which had been coated with Matrigel with 50,000 cells in each well. Compounds were introduced each to five separate wells and incubated for 96 h. Media was changed after 48 h and at 96 h cells were fixed with 4% PFA and stained for insulin and glucagon as described above with the following modifications. Antibody incubations and washes were performed using a BioTek EL405 plate washer and dispenser. Cells were imaged with the Cellomics ArrayScan high-content imaging system. Nine fields of view were captured for each well and analyzed for cell number and fluorescent content. The percentage of cells expressing insulin and glucagon protein was calculated for each well.

### Electron microscopy

To analyze the granule ultrastructure, human islets or differentiated SC-alpha cells were fixed for 2 h at RT in fixative (2.5% glutaraldehyde, 1.25% PFA and 0.03% picric acid in 0.1 M sodium cacodylate buffer, pH 7.4). Subsequently, islets or SC-alpha cells were washed in 0.1 M cacodylate buffer and post-fixed with 1% Osmium tetroxide (OsO_4_)/1.5% potassium ferrocyanide (KFeCN_6_) for 1 h, washed 2× in water, 1× Maleate buffer (MB) and incubated in 1% uranyl acetate in MB for 1 h followed by two washes in water and subsequent dehydration in grades of alcohol (10 min each; 50%, 70%, 90%, 2 × 10 min 100%). The samples were then put in propylene oxide for 1 h and infiltrated overnight in a 1:1 mixture of propylene oxide and TAAB Epon (Marivac Canada Inc.). The following day, the samples were embedded in TAAB Epon and polymerized at 60 °C for 48 h.

### Cell transplantation

All animal studies were conducted with approval from appropriate institutional oversight committees: Mayo Clinic Institutional Animal Care and Use Committee; Harvard University/Faculty of Arts & Sciences (HU/FAS) Standing Committee on the Use of Animals in Research and Teaching, Local Ethics Review Committee for Animal Experiments (Gothenburg, Sweden). Pre-surgery, animals were housed in groups within sterile cages with unrestricted access to food and water. Ambient temperature was maintained between 18 and 25 °C, humidity 30−70% with 12 h light/dark cycles. Transplantation of cell clusters was performed as previously described^8^. Briefly, 5 × 10^6^ cells were injected under the kidney capsule of male SCID-beige mice. Control mice underwent a mock surgery where saline was injected into the kidney capsule. Post-surgery, mice were single housed and monitored for up to 8 weeks after transplantation. Kidney grafts were harvested at various time points and stained for insulin and glucagon as described above.

### Continuous glucose monitoring

Transplanted mice were monitored using a Dexcom G4 continuous glucose monitoring (CGM) system. Each mouse was anesthetized with isoflurane prior to application of the CGM sensor and transmitter. Sensors were inserted into the subcutaneous space on the back of the mice and attached using wound clips. The adhesive patch for each sensor was trimmed to approximately 5 mm around the sensor and wound clips were used to strengthen adhesion. Sensors were calibrated twice a day using a handheld glucometer (Accu-Chek) and tail bleeds. Sensors were reset (stopped and restarted) every 7 days throughout the study (4 weeks total). After reset, sensors went through a 2-h calibration period in which no interstitial glucose readings were collected. As has been previously described^65^ interstitial glucose readings were recorded and used to approximate blood glucose concentrations.

### Electrophysiology

Electrical activity was recorded from alpha cells in intact human pancreatic islets using methods similar to those previously established for mouse islets^39^. Measurements from SC-alpha cells were made essentially as described for dispersed human islet cells^37^. For both cell types, measurements were performed from metabolically intact cell using the perforated patch whole-cell technique.

### Data analysis and calculations

Data processing of the CGM-measured glucose concentrations was conducted using MATLAB 2015b (MathWorks). The ultradian period of the CGM-measured glucose rhythms was quantified using autocorrelation analysis as previously described for the characterization of pulsatile insulin secretion^42^. MATLAB 2015b was used to calculate correlation coefficients of the CGM-measured glucose concentrations. The dominant circadian period in glucose concentration was assessed using Lomb-Scargle periodogram analysis using ClockLab (Actimetrics).

### Statistics and data reproducibility

Statistical analysis was performed using either ANOVA or a paired or unpaired Student’s *t* test, where appropriate (GraphPad Prism v.8.0). Representative images from Figs. 1d, 3e and Supplementary Fig. S10e, f were performed in three biologically independent samples. Images from Fig. 5a−i and Supplementary Fig. S4 were performed on one sample and are representative of three separate images obtained per sample. Western blots performed in Supplementary Figs. S2c and S18 were performed once.

## Supplementary information

Supplementary Information

## Data Availability

Full scans of Western blots are shown in Supplementary Fig. 22. Raw and processed single-cell RNA sequencing data that support the findings of this study are available in the Gene Expression Omnibus under the accession number GSE138857. Human islet data were obtained from publicly available sources, GEO: GSE84133 and GEO: GSE114297. All other data are available from the corresponding authors upon reasonable request.

## Source data

Source Data

## Code availability

Code and instructions for the analysis of Indrops sequencing can be found at: https://github.com/indrops/indrops/ and https://github.com/lvdmaaten/bhtsne. Code used for analysis of 10X Chromium sequencing data can be found at: https://satijalab.org/seurat/ and http://cole-trapnell-lab.github.io/monocle-release/docs/.

## References

[1] Brissova M (2018). Alpha cell function and gene expression are compromised in type 1 diabetes. Cell Rep..

[2] Unger RH, Cherrington AD (2012). Glucagonocentric restructuring of diabetes: a pathophysiologic and therapeutic makeover. J. Clin. Invest..

[3] Leckie AM, Graham MK, Grant JB, Ritchie PJ, Frier BM (2005). Frequency, severity, and morbidity of hypoglycemia occurring in the workplace in people with insulin-treated diabetes. Diabetes Care.

[4] Jiang G, Zhang BB (2003). Glucagon and regulation of glucose metabolism. Am. J. Physiol. Endocrinol. Metab..

[5] Mutel E (2011). Control of blood glucose in the absence of hepatic glucose production during prolonged fasting in mice: induction of renal and intestinal gluconeogenesis by glucagon. Diabetes.

[6] Fanne RA (2011). Neuroprotection by glucagon: role of gluconeogenesis. J. Neurosurg..

[7] Siafarikas A (2012). Early loss of the glucagon response to hypoglycemia in adolescents with type 1 diabetes. Diabetes Care.

[8] Pagliuca FW (2014). Generation of functional human pancreatic beta cells in vitro. Cell.

[9] Russ HA (2015). Controlled induction of human pancreatic progenitors produces functional beta-like cells in vitro. EMBO J..

[10] Rezania A (2014). Reversal of diabetes with insulin-producing cells derived in vitro from human pluripotent stem cells. Nat. Biotechnol..

[11] Veres A (2019). Charting cellular identity during human in vitro beta-cell differentiation. Nature.

[12] Spijker HS (2013). Conversion of mature human beta-cells into glucagon-producing alpha-cells. Diabetes.

[13] van der Meulen T, Huising MO (2015). Role of transcription factors in the transdifferentiation of pancreatic islet cells. J. Mol. Endocrinol..

[14] Piran R (2014). Pharmacological induction of pancreatic islet cell transdifferentiation: relevance to type I diabetes. Cell Death Dis..

[15] Rezania A (2011). Production of functional glucagon-secreting alpha-cells from human embryonic stem cells. Diabetes.

[16] Kelly OG (2011). Cell-surface markers for the isolation of pancreatic cell types derived from human embryonic stem cells. Nat. Biotechnol..

[17] Bruin JE (2014). Characterization of polyhormonal insulin-producing cells derived in vitro from human embryonic stem cells. Stem Cell Res..

[18] van der Meulen T, Huising MO (2014). Maturation of stem cell-derived beta-cells guided by the expression of urocortin 3. Rev. Diabet. Stud..

[19] Rezania A (2013). Enrichment of human embryonic stem cell-derived NKX6.1-expressing pancreatic progenitor cells accelerates the maturation of insulin-secreting cells in vivo. Stem Cells.

[20] Riopel M, Li J, Fellows GF, Goodyer CG, Wang R (2014). Ultrastructural and immunohistochemical analysis of the 8−20 week human fetal pancreas. Islets.

[21] Lee YS, Lee C, Choung JS, Jung HS, Jun HS (2018). Glucagon-like peptide 1 increases beta-cell regeneration by promoting alpha- to beta-cell transdifferentiation. Diabetes.

[22] D’Amour KA (2006). Production of pancreatic hormone-expressing endocrine cells from human embryonic stem cells. Nat. Biotechnol..

[23] Md Moin AS (2016). Increased frequency of hormone negative and polyhormonal endocrine cells in lean individuals with type 2 diabetes. J. Clin. Endocrinol. Metab..

[24] Md Moin AS (2016). Increased hormone-negative endocrine cells in the pancreas in type 1 diabetes. J. Clin. Endocrinol. Metab..

[25] Riedel MJ (2012). Immunohistochemical characterisation of cells co-producing insulin and glucagon in the developing human pancreas. Diabetologia.

[26] Klein AM (2015). Droplet barcoding for single-cell transcriptomics applied to embryonic stem cells. Cell.

[27] Baron M (2016). A single-cell transcriptomic map of the human and mouse pancreas reveals inter- and intra-cell population structure. Cell Syst..

[28] Hashimoto T (1988). Transient coappearance of glucagon and insulin in the progenitor cells of the rat pancreatic islets. Anat. Embryol..

[29] Alvarez-Dominguez JR (2020). Circadian entrainment triggers maturation of human in vitro islets. Cell Stem Cell.

[30] Osafune K (2008). Marked differences in differentiation propensity among human embryonic stem cell lines. Nat. Biotechnol..

[31] Pfeifer CR (2015). Quantitative analysis of mouse pancreatic islet architecture by serial block-face SEM. J. Struct. Biol..

[32] MacDonald PE (2007). A K ATP channel-dependent pathway within alpha cells regulates glucagon release from both rodent and human islets of Langerhans. PLoS Biol..

[33] Vieira E, Salehi A, Gylfe E (2007). Glucose inhibits glucagon secretion by a direct effect on mouse pancreatic alpha cells. Diabetologia.

[34] Gromada J, Franklin I, Wollheim CB (2007). Alpha-cells of the endocrine pancreas: 35 years of research but the enigma remains. Endocr. Rev..

[35] Salehi A, Vieira E, Gylfe E (2006). Paradoxical stimulation of glucagon secretion by high glucose concentrations. Diabetes.

[36] Dhalla AK (2014). Blockade of Na+ channels in pancreatic alpha-cells has antidiabetic effects. Diabetes.

[37] Ramracheya R (2010). Membrane potential-dependent inactivation of voltage-gated ion channels in alpha-cells inhibits glucagon secretion from human islets. Diabetes.

[38] Cejvan K, Coy DH, Holst JJ, Cerasi E, Efendic S (2002). Gliclazide directly inhibits arginine-induced glucagon release. Diabetes.

[39] Zhang Q (2013). Role of KATP channels in glucose-regulated glucagon secretion and impaired counterregulation in type 2 diabetes. Cell Metab..

[40] Korstanje R (2017). Continuous glucose monitoring in female NOD mice reveals daily rhythms and a negative correlation with body temperature. Endocrinology.

[41] Qian J, Yeh B, Rakshit K, Colwell CS, Matveyenko AV (2015). Circadian disruption and diet-induced obesity synergize to promote development of beta-cell failure and diabetes in male rats. Endocrinology.

[42] Lang DA, Matthews DR, Peto J, Turner RC (1979). Cyclic oscillations of basal plasma glucose and insulin concentrations in human beings. N. Engl. J. Med..

[43] Sharma A (2017). Glucose metabolism during rotational shift-work in healthcare workers. Diabetologia.

[44] Calbet JA, MacLean DA (2002). Plasma glucagon and insulin responses depend on the rate of appearance of amino acids after ingestion of different protein solutions in humans. J. Nutr..

[45] Claessens M, Saris WH, van Baak MA (2008). Glucagon and insulin responses after ingestion of different amounts of intact and hydrolysed proteins. Br. J. Nutr..

[46] Johnston NR (2016). Beta cell hubs dictate pancreatic islet responses to glucose. Cell Metab..

[47] Rorsman P, Huising MO (2018). The somatostatin-secreting pancreatic delta-cell in health and disease. Nat. Rev. Endocrinol..

[48] Rodriguez-Diaz R (2018). Paracrine interactions within the pancreatic islet determine the glycemic set point. Cell Metab..

[49] Ye L, Robertson MA, Hesselson D, Stainier DY, Anderson RM (2015). Glucagon is essential for alpha cell transdifferentiation and beta cell neogenesis. Development.

[50] Cryer PE (2008). The barrier of hypoglycemia in diabetes. Diabetes.

[51] De Marinis YZ (2010). Enhancement of glucagon secretion in mouse and human pancreatic alpha cells by protein kinase C (PKC) involves intracellular trafficking of PKCalpha and PKCdelta. Diabetologia.

[52] Brunton S (2014). GLP-1 receptor agonists vs. DPP-4 inhibitors for type 2 diabetes: is one approach more successful or preferable than the other?. Int. J. Clin. Pr..

[53] Teitelman G, Alpert S, Polak JM, Martinez A, Hanahan D (1993). Precursor cells of mouse endocrine pancreas coexpress insulin, glucagon and the neuronal proteins tyrosine hydroxylase and neuropeptide Y, but not pancreatic polypeptide. Development.

[54] De Krijger RR (1992). The midgestational human fetal pancreas contains cells coexpressing islet hormones. Dev. Biol..

[55] Yamamoto K (2017). Protein kinase C-delta signaling regulates glucagon secretion from pancreatic islets. J. Med. Invest..

[56] Furstenau U, Schwaninger M, Blume R, Kennerknecht I, Knepel W (1997). Characterization of a novel protein kinase C response element in the glucagon gene. Mol. Cell Biol..

[57] Barnett ME, Madgwick DK, Takemoto DJ (2007). Protein kinase C as a stress sensor. Cell Signal.

[58] Termini CM, Gillette JM (2017). Tetraspanins function as regulators of cellular signaling. Front. Cell Dev. Biol..

[59] Slater SJ (1995). Direct activation of protein kinase C by 1 alpha,25-dihydroxyvitamin D3. J. Biol. Chem..

[60] Zilionis R (2017). Single-cell barcoding and sequencing using droplet microfluidics. Nat. Protoc..

[61] Wolf FA, Angerer P, Theis FJ (2018). SCANPY: large-scale single-cell gene expression data analysis. Genome Biol..

[62] Butler A, Hoffman P, Smibert P, Papalexi E, Satija R (2018). Integrating single-cell transcriptomic data across different conditions, technologies, and species. Nat. Biotechnol..

[63] Stuart T (2019). Comprehensive integration of single-cell data. Cell.

[64] Trapnell C (2014). The dynamics and regulators of cell fate decisions are revealed by pseudotemporal ordering of single cells. Nat. Biotechnol..

[65] Klueh U (2006). Continuous glucose monitoring in normal mice and mice with prediabetes and diabetes. Diabetes Technol. Ther..

