# Supplementary material for: A method for the generation of human stem cell-derived alpha cells
Source: Nat Commun. 2020 May 7;11:2241. doi: 10.1038/s41467-020-16049-3 (PMC7205884; doi:10.1038/s41467-020-16049-3)
Supplement: Supplementary file 1 — Supplementary Information [file 41467_2020_16049_MOESM1_ESM.pdf]

A method for the generation of human stem cell-derived alpha cells

Peterson et al.

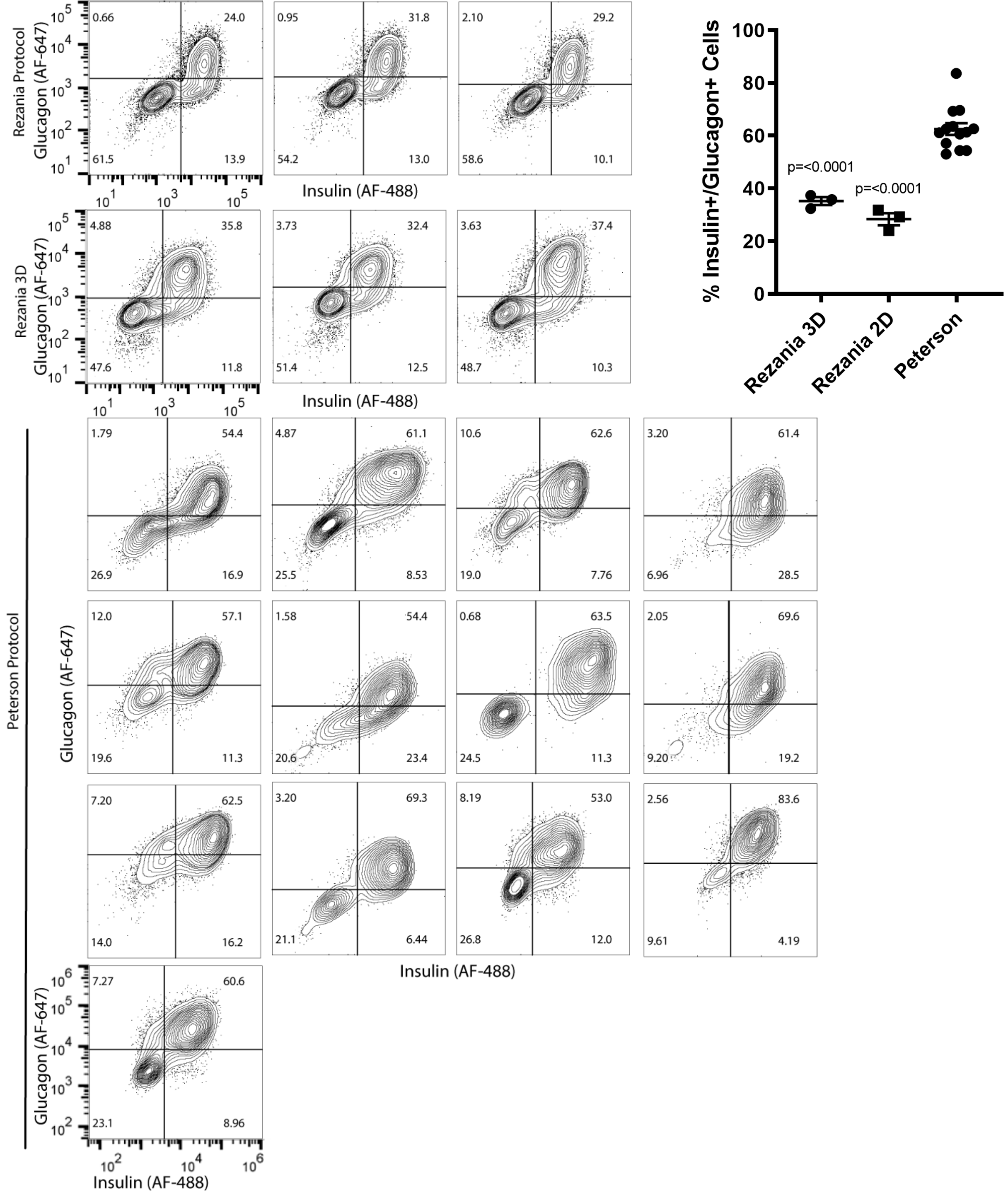

Supplemental Figure 1. Flow cytometry plots for differentiations at the end of stage 5 showing the percentage of polyhormonal (Ins+/Gcg+) cells from the original Rezania protocol (top, n=3 biologically independent samples), the Rezania protocol in 3D format (middle, n=3 biologically independent samples) and the Peterson protocol (bottom, n=13 biologically independent samples). Percentage of polyhormonal cells is summarized in the graph. Averaged data is expressed as mean +/- SEM. Significance calculated using an Ordinary one-way ANOVA with Dunnett multiple comparison test.

Supplemental Figure 2

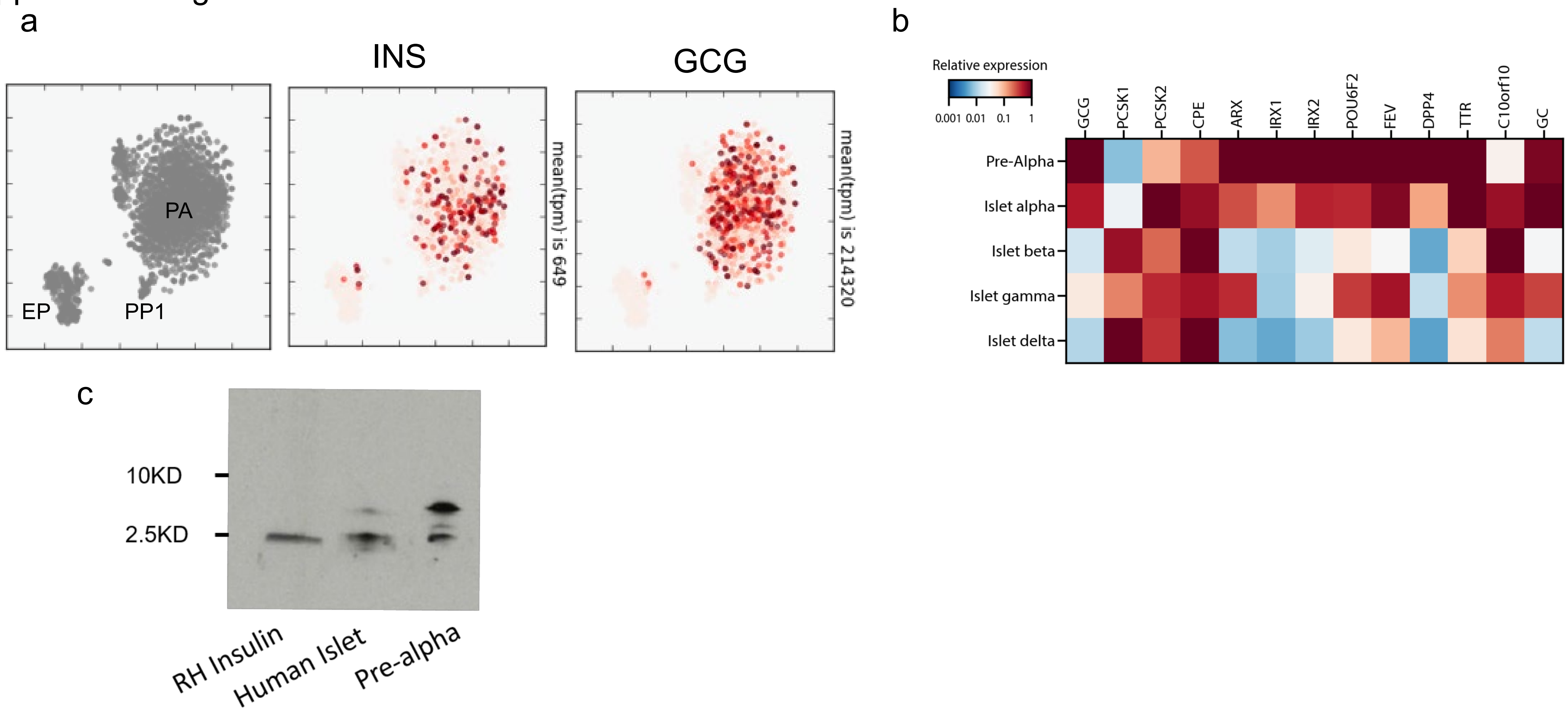

Supplemental Figure 2. a) tSNE plot of cells showing expression of insulin and glucagon in pre-alpha cells. b) Heatmap of key alpha cell marker transcript expression levels in pre-alpha cells compared to endocrine cell types from human islets. c) Western blot shows the identification of insulin content in pre-alpha cells compared to recombinant human (RH) insulin and human islets.

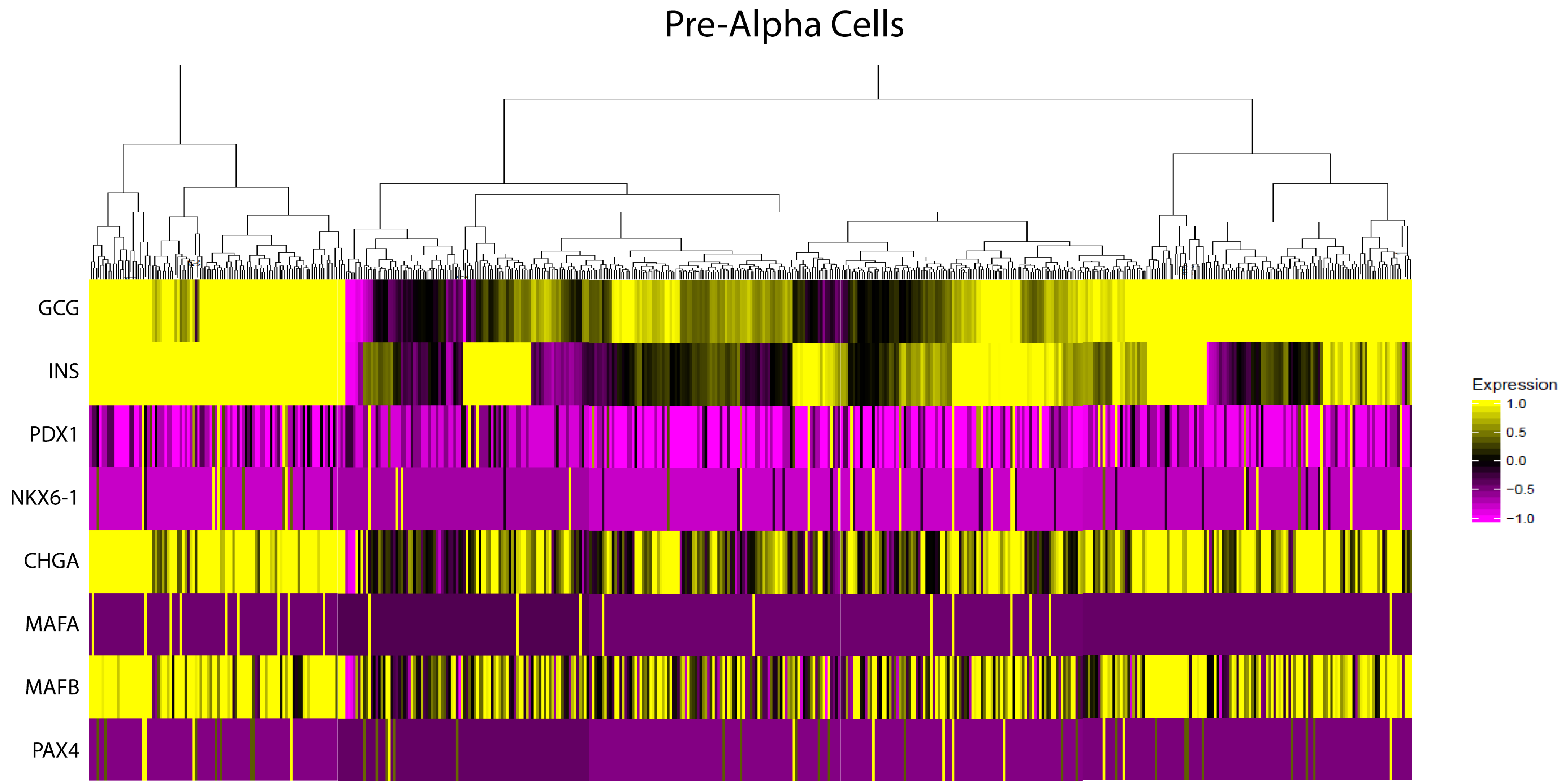

Supplemental Figure 3. Hierarchical heatmap of common alpha (GCG), alpha and beta (CHGA, MAFB) and beta (INS, PDX1, NKX6-1, MAFA, PAX4) cell markers in pre-alpha cells. Columns correspond to individual cells and rows correspond to individual genes. Color scale based on *z*-score distribution from −1 (purple) to 1 (yellow).

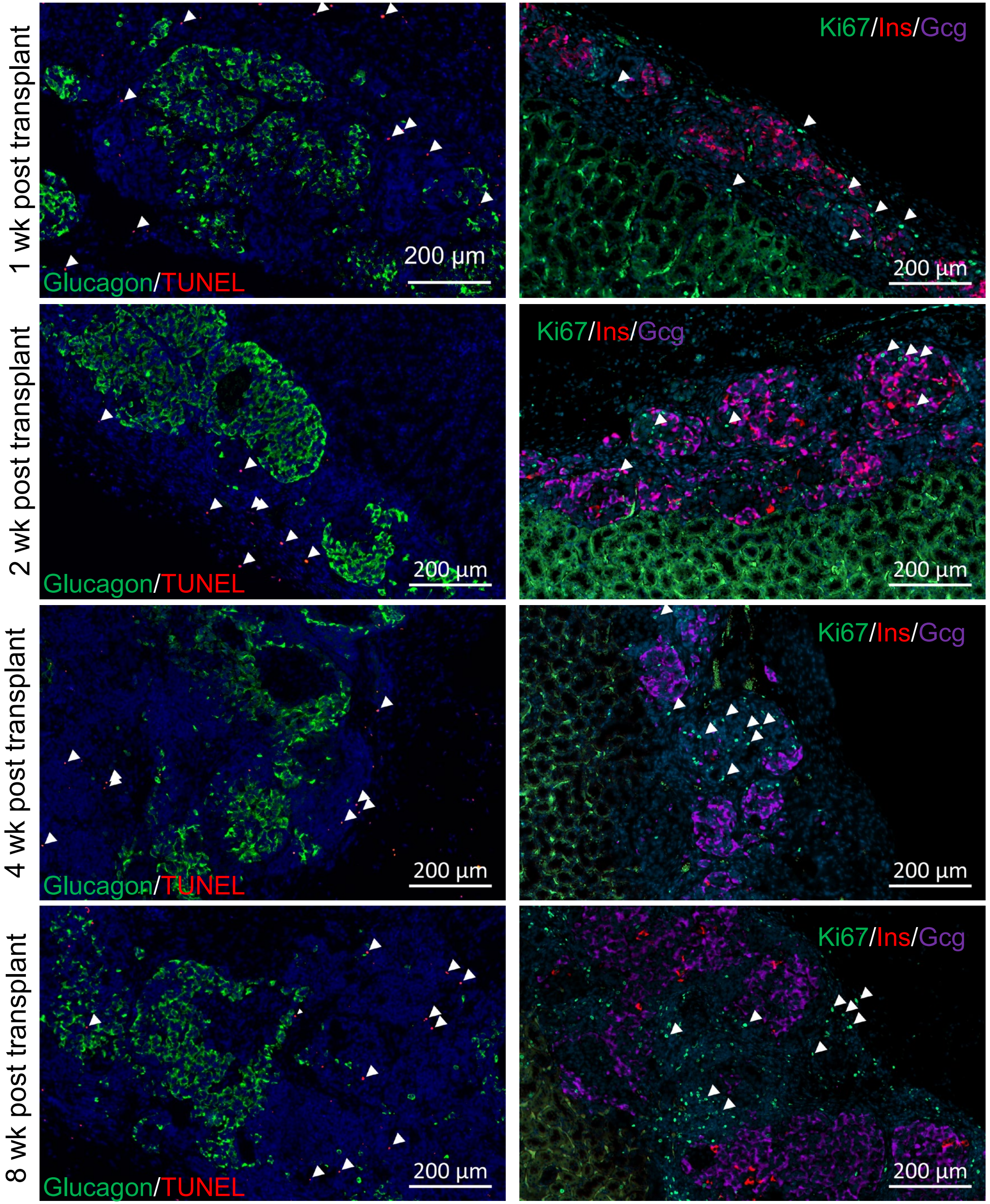

Supplemental Figure 4.
Characterization of TUNEL and Ki67 in transplanted pre-alpha cells at 1 week, 2 weeks, 4 weeks and 8 weeks post transplant. TUNEL positive cells are only occasionally observed colocalizing with glucagon. Ki67 positive cells are occasionally found colocalizing with glucagon at all time points, but are mainly restricted to non-hormone positive cells. Arrows indicate TUNEL positive cells (Left) or Ki67 positive cells (Right)

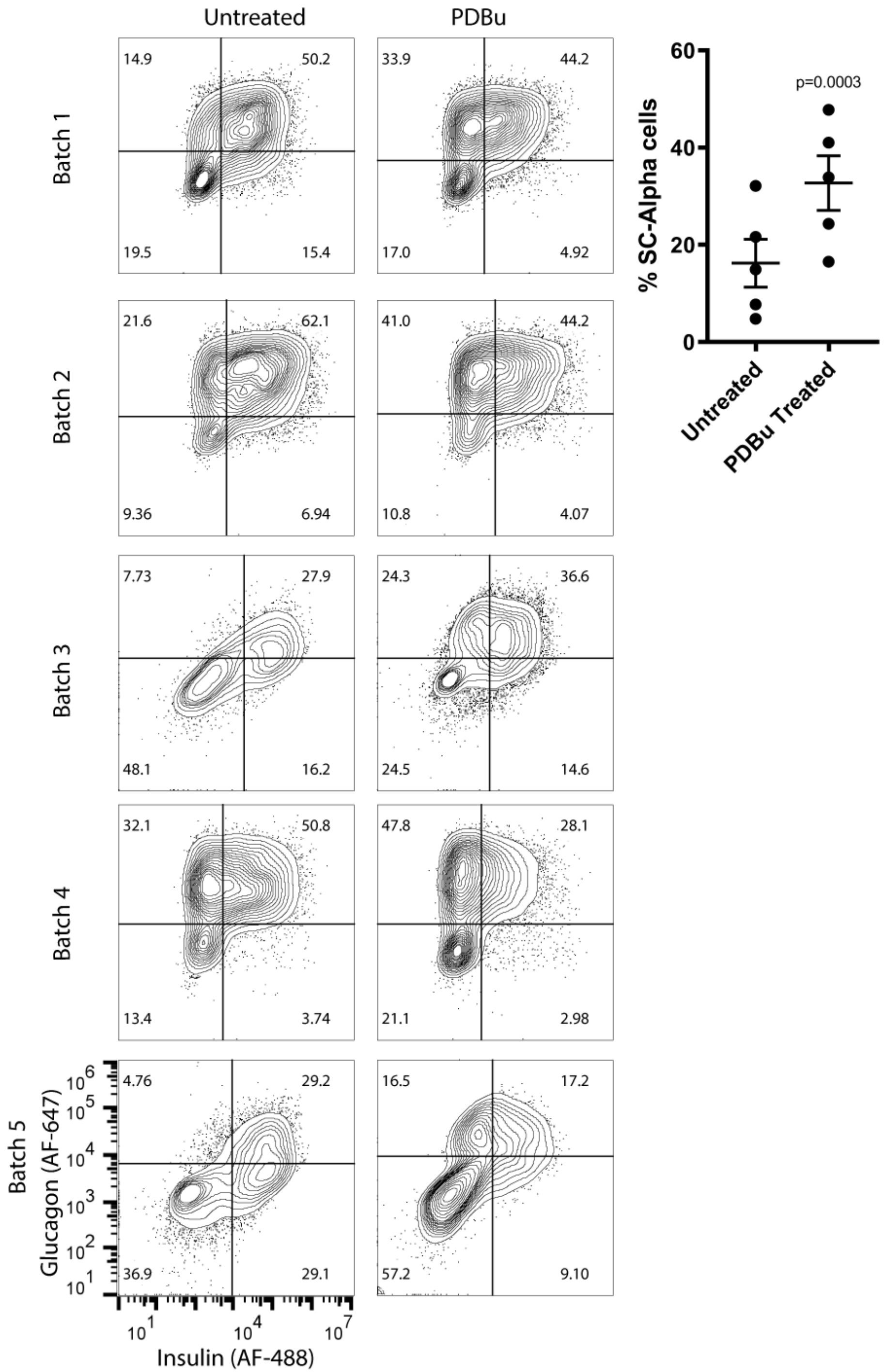

Supplemental Figure 5. Flow cytometry plots for differentiations at the end of stage 6 performed with and without treatment of PDBu during stage 6 and showing the percentage of monohormonal (Ins-/Gcg+) cells for each differentiation (n=5 biologically independent samples). Percentage of SC-alpha cells is summarized in the graph. Averaged data is expressed as mean +/- SEM. Significance calculated using a paired two-sided Student's t-test.

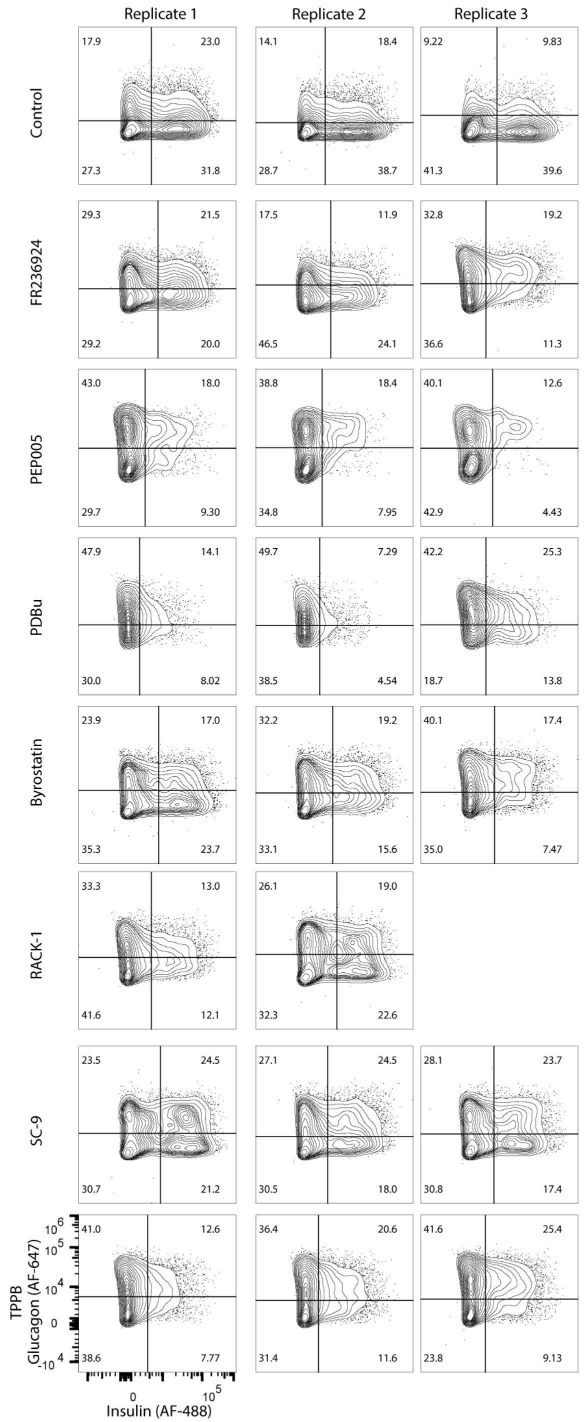

Supplemental Figure 6. Flow cytometry plots for differentiations performed with various PKC activators and analyzed at the end of stage 6. Flow plots show the percentage of monohormonal (Ins-/Gcg+) SC-alpha cells for each compound (n=3 biologically independent samples for most conditions). Percentage of polyhormonal cells is summarized in Figure 3.

Supplemental Figure 7

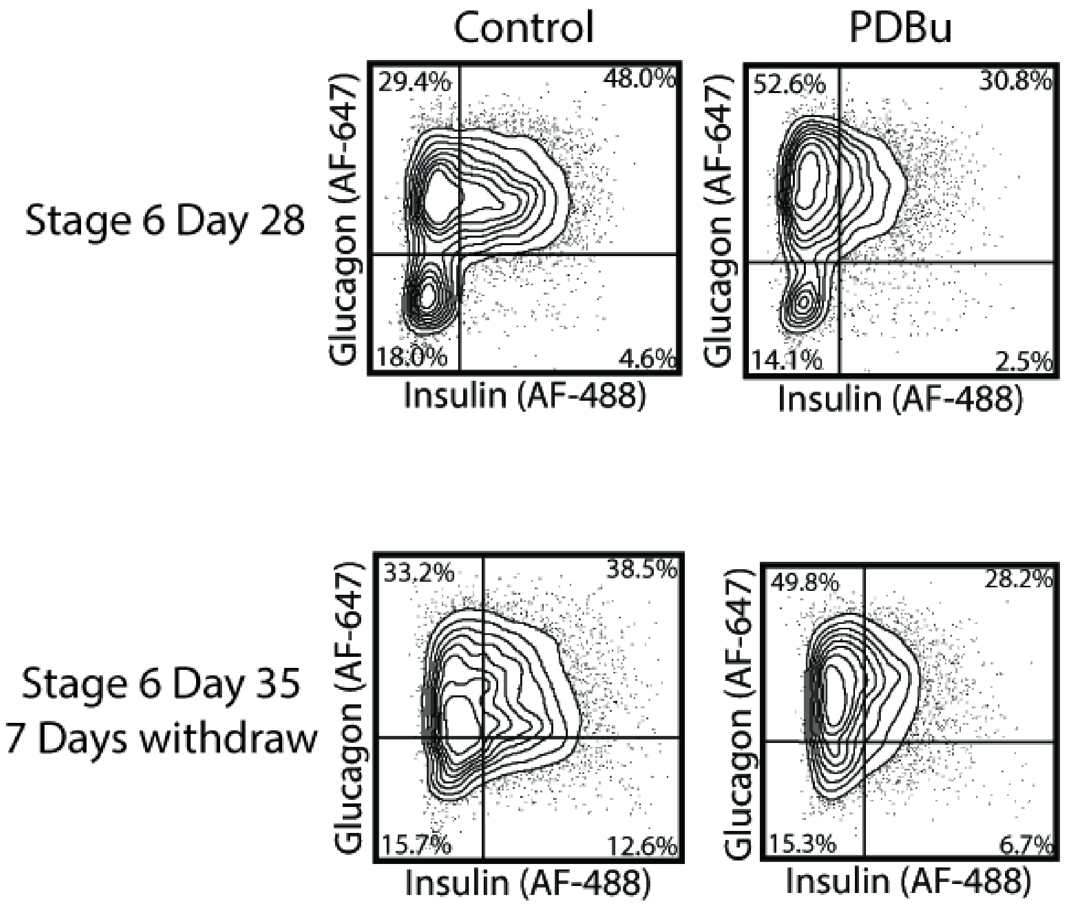

Supplemental Figure 7. Withdrawal of PDBu. SC-alpha cells derived with PDBu and control conditions were cultured in the absence of PDBu for 7 days to determine the stability of the monohormonal phenotype. Withdrawal of PDBu for 7 days did not significantly reduce the percentage of monohormonal SC-alpha cells.

Supplemental Figure 8

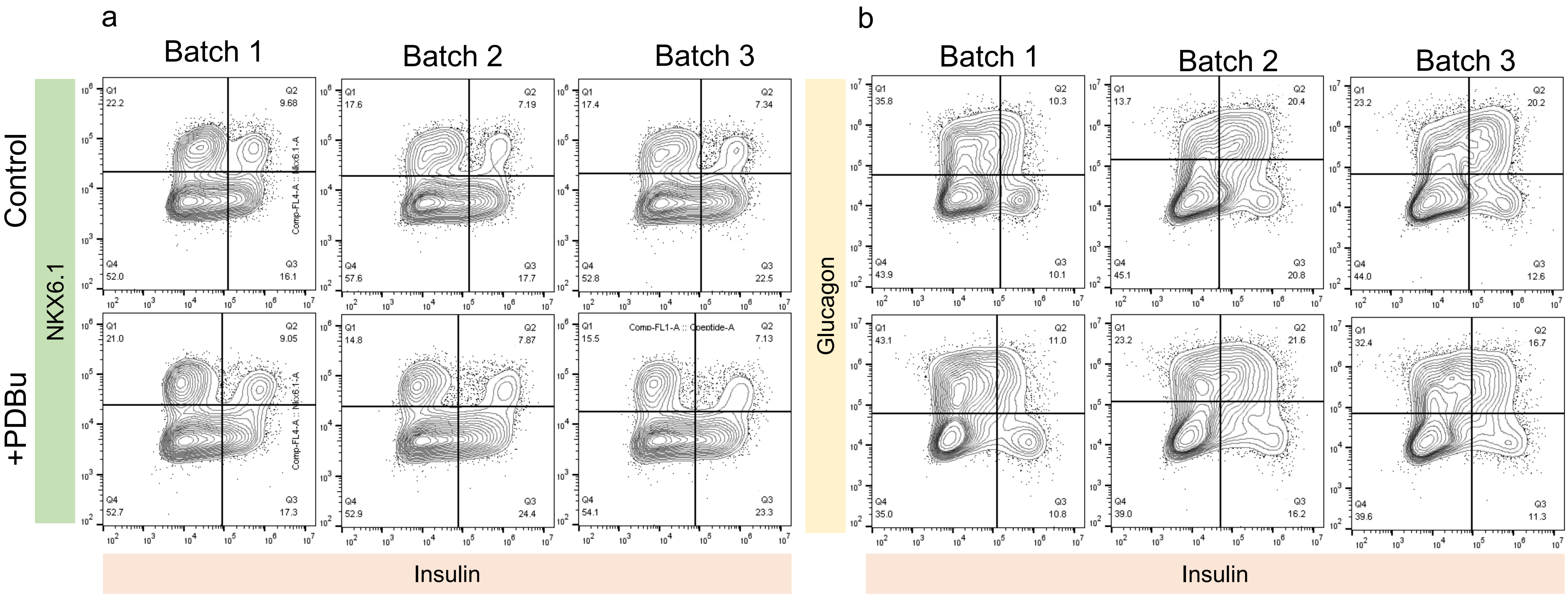

Supplemental Figure 8. Flow cytometry of three separate batches of HUES8 cells differentiated to SC-beta cells and treated with PDBu for 14 days during stage 6. a) PDBu treatment did not effect the percentage of NKX6.1/Insulin co-positive cells. b) PDBu treatment increases the percentage of glucagon monohormonal cells.

Supplemental Figure 9

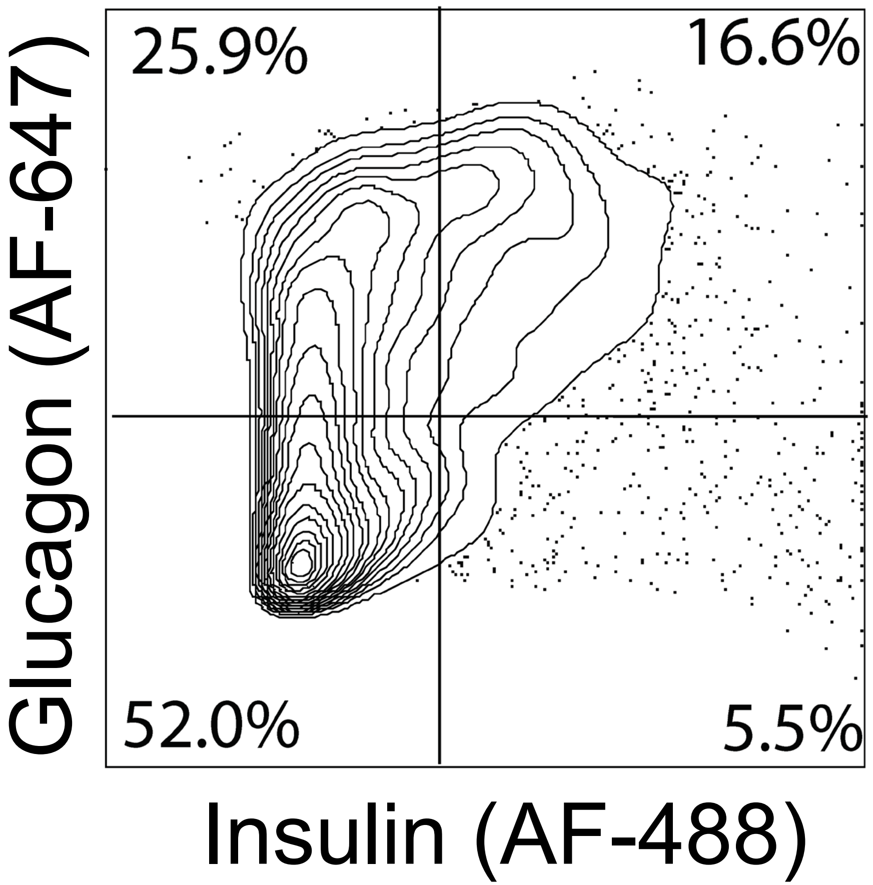

Supplemental Figure 9. Flow cytometry of the iPS cell line 1016 differentiated with the SC-alpha cell protocol. An increased concentration of LDN (1µM) during the differentiation (day 6, 7 and 8) was used and resulted in better differentiations in the 1016 cell line.

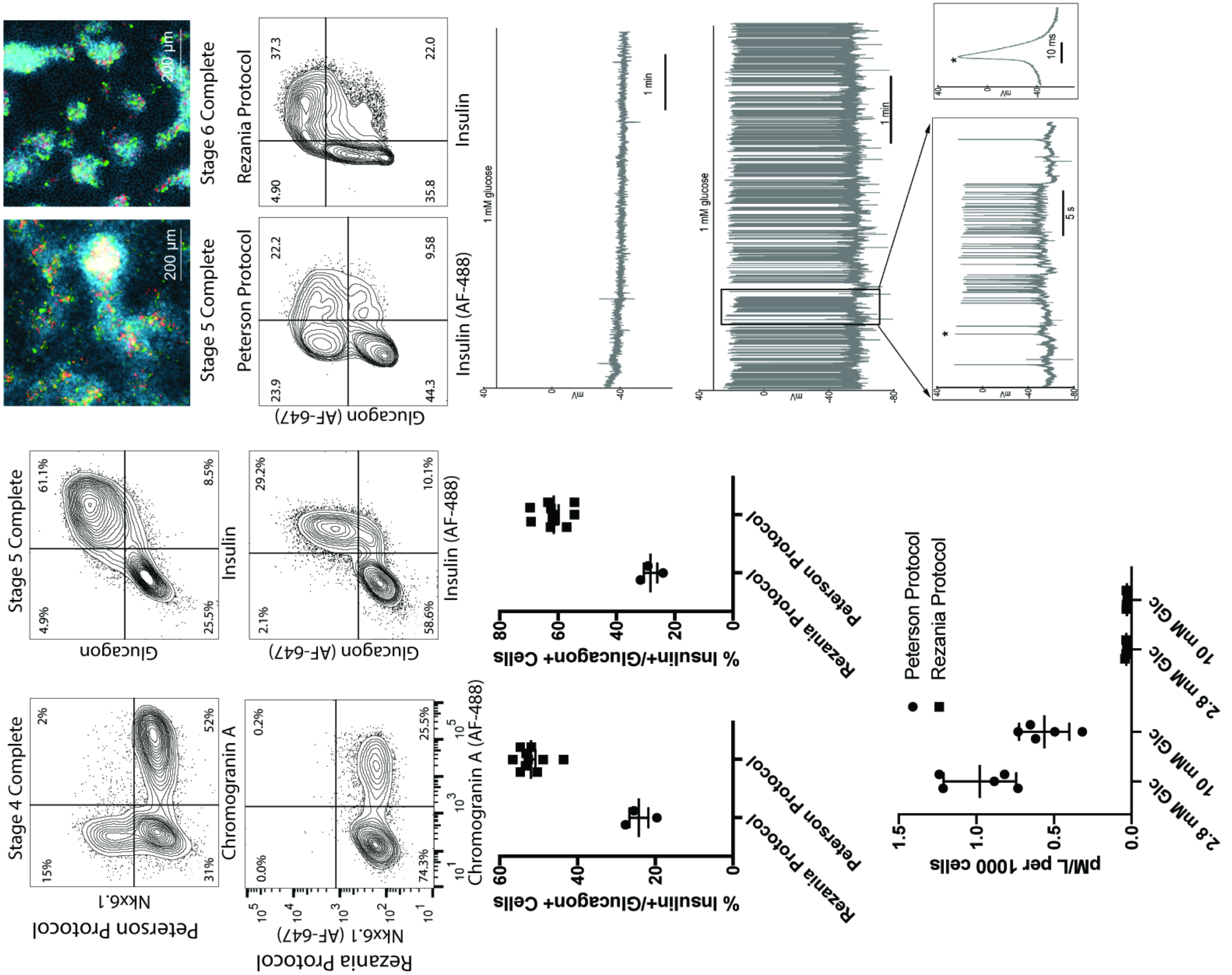

Supplemental Figure 10. Head-to-head comparison of Peterson and Rezania protocols in HUES8 cell line.
a) Representative flow cytometry results for cell populations at the end of stage 4, 5 and 6 for both the Peterson and Rezania protocols. Key markers were evaluated (Nkx6.1 and Chromogranin A for stage 4 complete, insulin and glucagon for stage 5 and 6 complete). Summary of multiple differentiations is shown in b) for stage 4 (Rezania n=3, Peterson n=10), c) for stage 5 (Rezania n=3, Peterson n=13) and d) for stage 6 (Rezania n=3, Peterson n=13). (Averaged data is expressed as mean +/- SEM). Individual replicate flow plots are shown in Supplemental figure 1, 11 and 12. The Rezania protocol results in scarce islands of hormone positive cells at stage 5 and stage 6 complete (e and f). These islands bud off the surface of the plate during the transition from stage 5 to stage 6, resulting in cell loss. g) comparison of glucagon secretion from stage 6 complete cells generated by Peterson (n=5) and Rezania (n=5) protocols in the 1016 iPS cell line at low and high glucose. (Averaged data is expressed as mean +/- SEM) h) Representative electrical activity recording of SC-alpha cell generated by the Peterson protocol in 1016 iPS cell line. Spontaneous action potentials are observed at low glucose (n=30). i) Representative electrical activity recording of glucagon positive cell generated by the Rezania protocol showing no electrical activity (n=4).

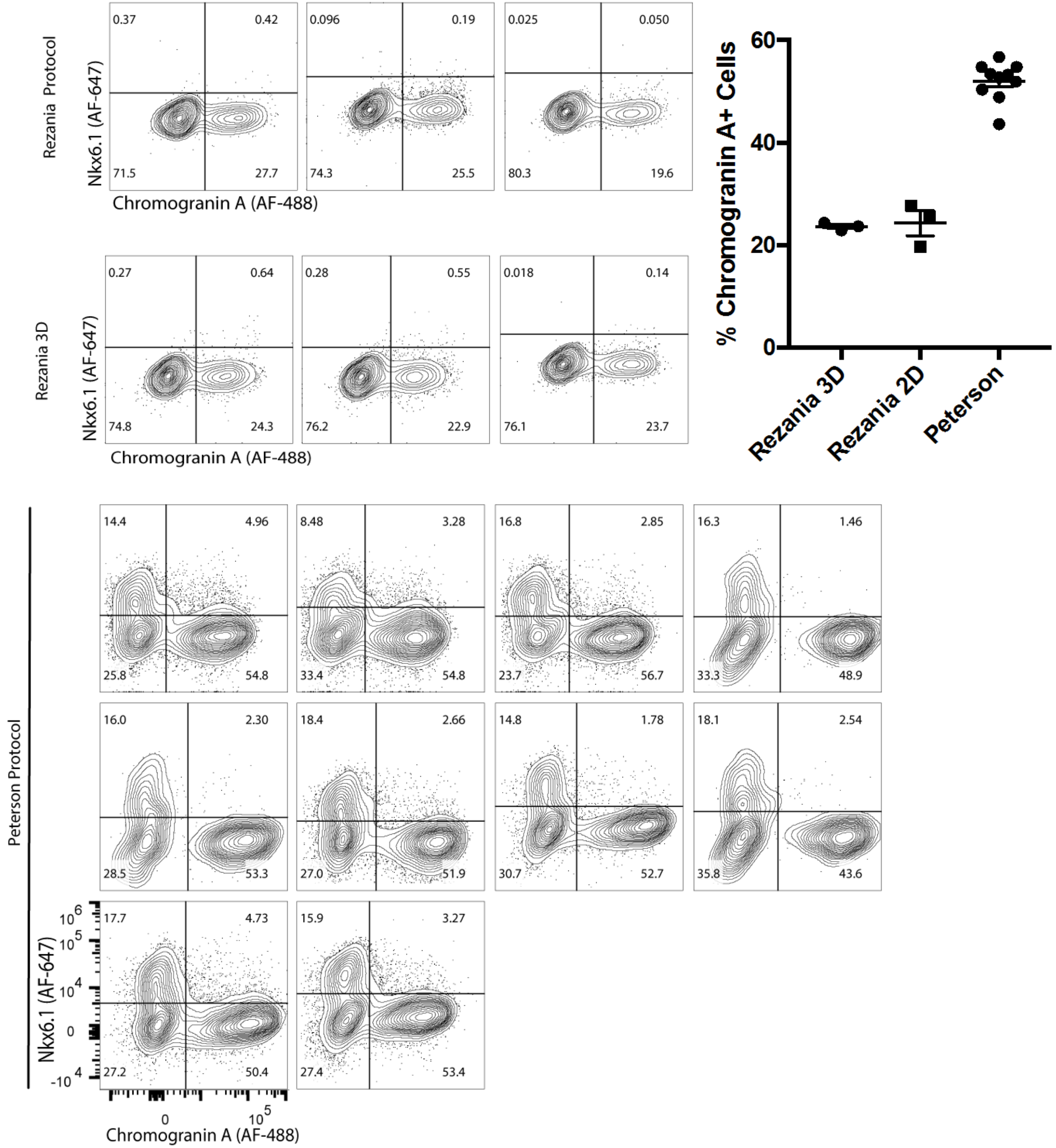

Supplemental Figure 11. Flow cytometry plots for differentiations at the end of stage 4 showing the percentage of chromogranin A+ cells for the original Rezania protocol (top, n=3 biologically independent samples), the Rezania protocol in 3D format (middle, n=3 biologically independent samples) and the Peterson protocol (bottom, n=10 biologically independent samples). Percentage of chromogranin A+ cells is summarized in the graph. Averaged data is expressed as mean +/- SEM.

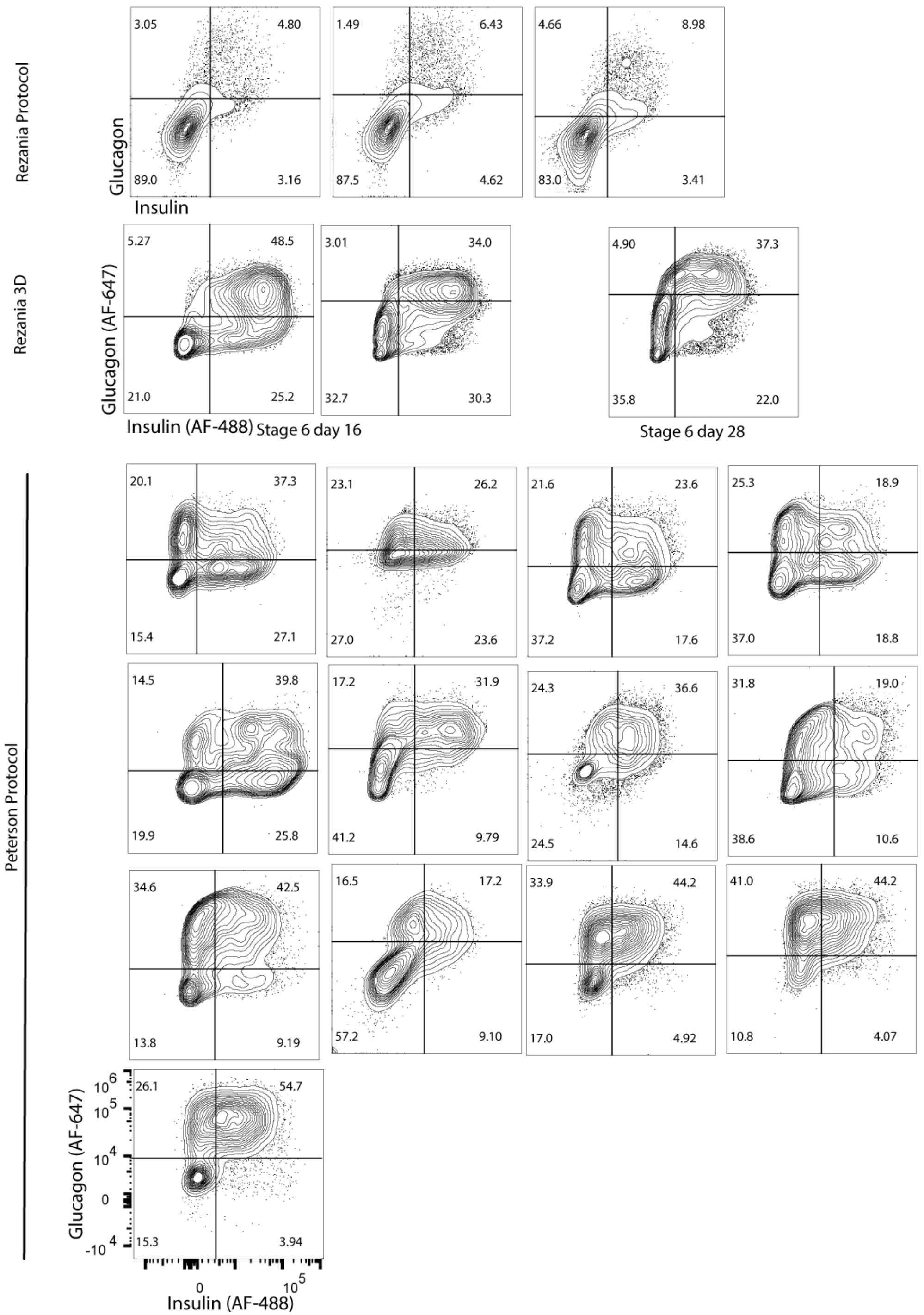

Supplemental Figure 12. Flow cytometry plots for differentiations at the end of stage 6 showing the percentage of monohormonal (Ins-/Gcg+) cells for the original Rezania protocol (top, n=3 biologically independent samples), the Rezania protocol in 3D format at stage 6 day 16 (n=2 biologically independent samples) and at stage 6 day 28 (n=1) (middle), and the Peterson protocol (bottom, n=13 biologically independent samples). Percentage of SC-alpha cells is summarized in Supplemental Figure 10d.

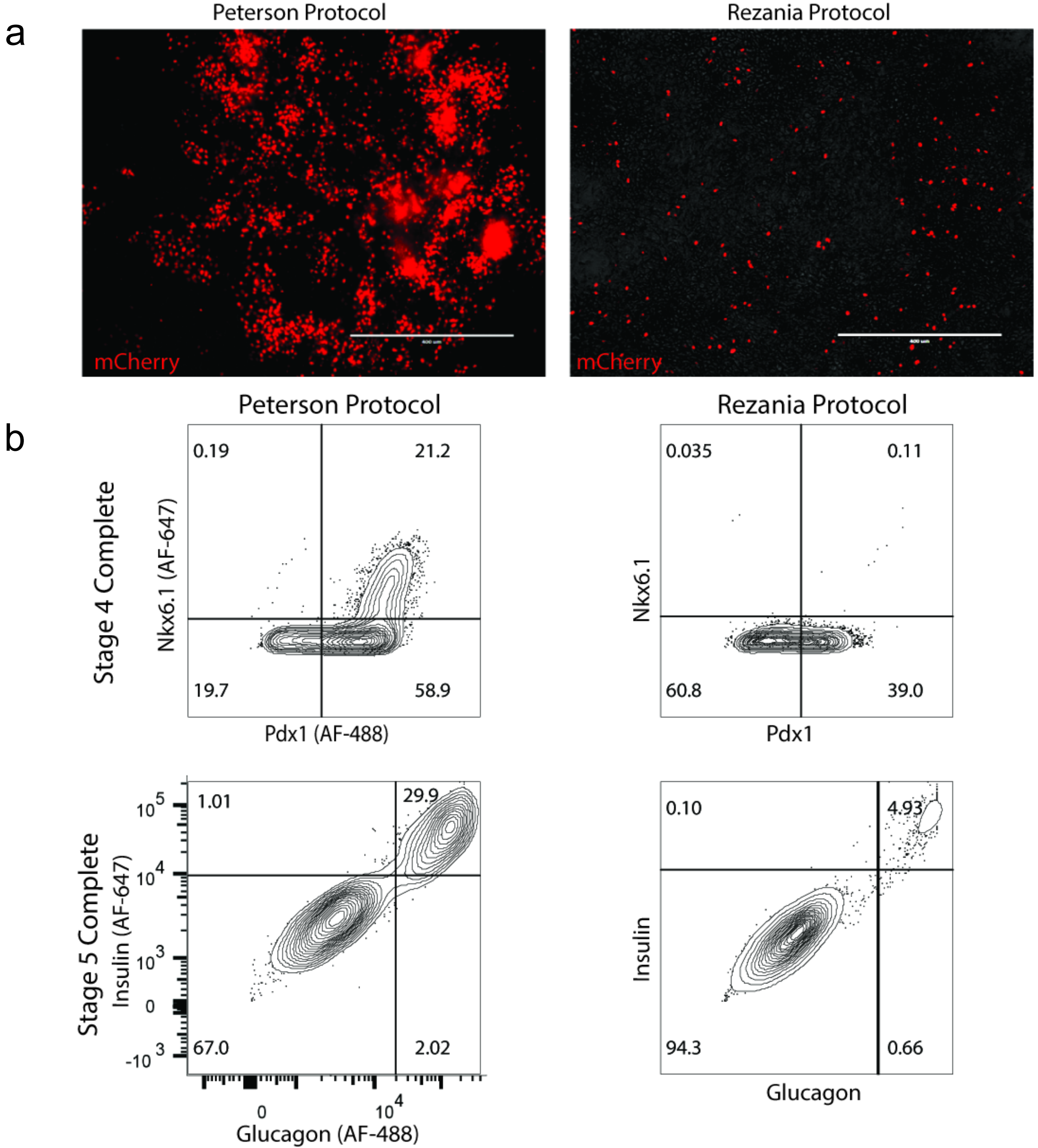

Supplemental Figure 13. Head-to-head comparison of Peterson and Rezania protocols in the 1016 cell line. a) Fluorescent images of cell population at stage 6 complete showing glucagon positive cells for both the Rezania and Peterson protocols. b) Representative flow cytometry results for cell populations at the end of stage 4 and 5 for both the Peterson and Rezania protocols.

Supplemental Figure 14

KEGG

Term	Pre-Alpha: Identified/Total, %	SC-Alpha: Identified/Total, %
Thermogenesis	17/229 (7.4%)	15/229 (6.6%)
Parkinson disease	14/142 (9.9%)	11/142 (7.7%)
Alzheimer disease	15/171 (8.8%)	12/171 (7.0%)
Oxidative phosphorylation	12/133 (9.0%)	11/133 (8.3%)
Non-alcoholic fatty liver disease	12/149 (8.1%)	9/149 (6.0%)
Huntington disease	13/193 (6.7%)	10/193 (5.2%)
Cardiac muscle contraction	8/78 (10.3%)	5/78 (6.4%)
Protein processing in endoplasmic reticulum	10/165 (6.1%)	9/165 (5.5%)
Estrogen signaling pathway	9/137 (6.6%)	9/137 (6.6%)
Fluid shear stress and atherosclerosis	9/139 (6.5%)	9/139 (6.5%)
Oxytocin signaling pathway	9/152 (5.9%)	9/152 (5.9%)
Gastric acid secretion	6/75 (8.0%)	6/75 (8.0%)
Pertussis	6/76 (7.9%)	6/76 (7.9%)
Amphetamine addiction	5/68 (7.4%)	5/68 (7.4%)
PPAR signaling pathway	5/74 (6.8%)	2/74 (2.7%)
Antigen processing and presentation	5/77 (6.5%)	7/77 (9.1%)
Necroptosis	7/162 (4.3%)	7/162 (4.3%)
cGMP-PKG signaling pathway	7/163 (4.3%)	5/163 (3.1%)
Protein export	3/23 (13.0%)	3/23 (13.0%)
Salivary secretion	5/90 (5.6%)	4/90 (4.4%)
Pathogenic Escherichia coli infection	4/55 (7.3%)	4/55 (7.3%)
IL-17 signaling pathway	5/93 (5.4%)	6/93 (6.5%)
Calcium signaling pathway	7/186 (3.8%)	4/186 (2.2%)
cAMP signaling pathway	7/199 (3.5%)	6/199 (3.0%)
Rap1 signaling pathway	7/206 (3.4%)	8/206 (3.9%)
Human immunodeficiency virus 1 infection	7/212 (3.3%)	9/212 (4.2%)
Arrhythmogenic right ventricular cardiomyopathy	4/72 (5.6%)	4/72 (5.6%)
Thyroid hormone synthesis	4/74 (5.4%)	4/74 (5.4%)
Neurotrophin signaling pathway	5/119 (4.2%)	4/119 (3.4%)
Tight junction	6/170 (3.5%)	6/170 (3.5%)
Ferroptosis	3/40 (7.5%)	2/40 (5.0%)
Complement and coagulation cascades	4/79 (5.1%)	3/79 (3.8%)
Type I diabetes mellitus	3/43 (7.0%)	3/43 (7.0%)
Dopaminergic synapse	5/131 (3.8%)	4/131 (3.1%)
Insulin secretion	4/85 (4.7%)	3/85 (3.5%)
Salmonella infection	4/86 (4.7%)	4/86 (4.7%)
Type II diabetes mellitus	3/46 (6.5%)	2/46 (4.3%)
Apoptosis	5/136 (3.7%)	5/136 (3.7%)
Vibrio cholerae infection	3/50 (6.0%)	4/50 (8.0%)
Phagosome	5/152 (3.3%)	7/152 (4.6%)
Glucagon signaling pathway	4/103 (3.9%)	5/103 (4.9%)
Viral myocarditis	3/59 (5.2%)	4/59 (6.9%)
Parathyroid hormone synthesis, secretion and action	4/106 (3.8%)	5/106 (4.7%)
Kaposi sarcoma-associated herpesvirus infection	5/186 (2.7%)	6/186 (3.2%)

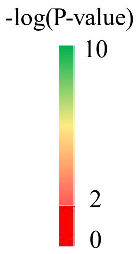

Supplemental Figure 14. Heatmap showing all pathways from KEGG terms for pre-alpha and SC-alpha cells. Columns show number of genes mapped to the pathway divided by number of possible genes in the pathway, in addition to its percentage. Scale is from –log(p-value) of 2 (light red) to 10 (green). All pathways that were not significant, i.e. –log(p-value) from 0 to 2, are labeled in red. P-values calculated using an EASE Score (modified Fisher Exact test).

GO

Term	Pre-Alpha: Identified/Total, %	SC-Alpha: Identified/Total, %
mitochondrial electron transport, cytochrome c to oxygen	6/20 (30%)	3/20 (2.1%)
cellular protein metabolic process	10/118 (8.5%)	11/118 (7.5%)
hydrogen ion transmembrane transport	8/61 (13.1%)	5/61 (3.4%)
G2/M transition of mitotic cell cycle	9/137 (6.6%)	7/137 (4.8%)
protein folding	10/180 (5.6%)	9/180 (6.2%)
regulation of insulin secretion	7/67 (10.4%)	3/67 (2.1%)
response to drug	12/304 (3.9%)	11/304 (7.5%)
substantia nigra development	6/49 (12.2%)	4/49 (2.7%)
generation of precursor metabolites and energy	6/53 (11.3%)	3/53 (2.1%)
transforming growth factor beta receptor signaling pathway	7/92 (7.6%)	7/92 (4.8%)
virion assembly	4/12 (33.3%)	4/12 (2.7%)
protein folding in endoplasmic reticulum	4/13 (30.8%)	3/13 (2.1%)
negative regulation of very-low-density lipoprotein particle remodeling	3/3 (100%)	0/3 (%)
platelet degranulation	7/103 (6.8%)	8/103 (5.5%)
reverse cholesterol transport	4/18 (22.2%)	1/18 (0.7%)
positive regulation of cyclic-nucleotide phosphodiesterase activity	3/5 (60%)	3/5 (2.1%)
protein stabilization	7/136 (5.1%)	6/136 (4.1%)
retinoid metabolic process	5/61 (8.2%)	2/61 (1.4%)
cell-cell adhesion	9/271 (3.3%)	8/271 (5.5%)
regulation of tumor necrosis factor-mediated signaling pathway	4/30 (13.3%)	4/30 (2.7%)
viral life cycle	4/31 (12.9%)	4/31 (2.7%)
ATF6-mediated unfolded protein response	3/9 (33.3%)	3/9 (2.1%)
lipoprotein biosynthetic process	3/9 (33.3%)	1/9 (0.7%)
positive regulation of ryanodine-sensitive calcium-release channel activity	3/9 (33.3%)	3/9 (2.1%)
response to cold	4/36 (11.1%)	4/36 (2.7%)
chaperone-mediated protein folding	4/37 (10.8%)	4/37 (2.7%)
Fc-epsilon receptor signaling pathway	7/178 (3.9%)	7/178 (4.8%)
ERBB2 signaling pathway	4/38 (10.5%)	4/38 (2.7%)
response to reactive oxygen species	4/39 (10.3%)	3/39 (2.1%)
regulation of necrotic cell death	3/12 (25%)	3/12 (2.1%)
positive regulation of DNA replication	4/42 (9.5%)	5/42 (3.4%)
MyD88-independent toll-like receptor signaling pathway	3/13 (23.1%)	3/13 (2.1%)
positive regulation of phosphoprotein phosphatase activity	3/13 (23.1%)	3/13 (2.1%)
regulation of type I interferon production	3/13 (23.1%)	3/13 (2.1%)
positive regulation of nitric oxide biosynthetic process	4/43 (9.3%)	4/43 (2.7%)
detection of calcium ion	3/14 (21.4%)	3/14 (2.1%)
phospholipid efflux	3/14 (21.4%)	0/14 (0%)
high-density lipoprotein particle remodeling	3/15 (20%)	0/15 (0%)
ion transmembrane transport	7/210 (3.3%)	7/210 (4.8%)
negative regulation of lipid catabolic process	3/16 (18.8%)	0/16 (0%)
regulation of release of sequestered calcium ion into cytosol by sarcoplasmic reticulum	3/18 (16.7%)	3/18 (2.1%)
response to corticosterone	3/18 (16.7%)	3/18 (2.1%)
negative regulation of apoptotic process	10/455 (2.2%)	15/455 (10.3%)
response to stress	4/61 (6.6%)	5/61 (3.4%)
response to nutrient	4/74 (5.4%)	5/74 (3.4%)
positive regulation of NF-kappaB transcription factor activity	5/133 (3.8%)	6/133 (4.1%)
positive regulation of cell differentiation	3/37 (8.1%)	4/37 (2.7%)
cellular response to calcium ion	3/51 (5.9%)	4/51 (2.7%)
negative regulation of translation	3/58 (5.2%)	5/58 (3.4%)
regulation of cell cycle	4/124 (3.2%)	6/124 (4.1%)
DNA damage response, signal transduction by p53 class mediator resulting in cell cycle arrest	3/62 (4.8%)	6/62 (4.1%)
protein refolding	2/15 (13.3%)	3/15 (2.1%)
negative regulation of transcription from RNA polymerase II promoter	10/720 (1.4%)	14/720 (9.6%)
translational elongation	2/18 (11.1%)	3/18 (2.1%)
cellular response to hormone stimulus	2/45 (4.4%)	4/45 (2.7%)
response to cytokine	2/52 (3.8%)	4/52 (2.7%)
positive regulation of translation	2/53 (3.8%)	4/53 (2.7%)
response to peptide hormone	1/44 (2.3%)	4/44 (2.7%)

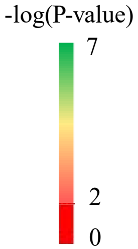

Supplemental Figure 15. Heatmap showing all pathways from GO-BP terms for pre-alpha and SC-alpha cells. Columns show number of genes mapped to the pathway divided by number of possible genes in the pathway, in addition to its percentage. Scale is from –log(p-value) of 2 (light red) to 7 (green). All pathways that were not significant, i.e. –log(p-value) from 0 to 2, are labeled in red. P-values calculated using an EASE Score (modified Fisher Exact test).

Supplemental Figure 16. Trajectory of pre-alpha cells into SC-alpha cells as determined by Monocle v.2. Top graph shows the pseudotime trajectory, labeled by cell type, with ordering determined by genes that co-vary with insulin. Bottom graph shows progression of cells over pseudotime from pre-alpha into SC-alpha cells. Each dot represents a single cell. Right: Heatmap of the top 50 genes which co-vary with the expression of insulin and are pseudotime-dependent. Columns correspond to individual cells and rows correspond to individual genes. Genes are clustered by expression profile. Color scale based on z-score distribution from -3 (blue) to 3 (red).

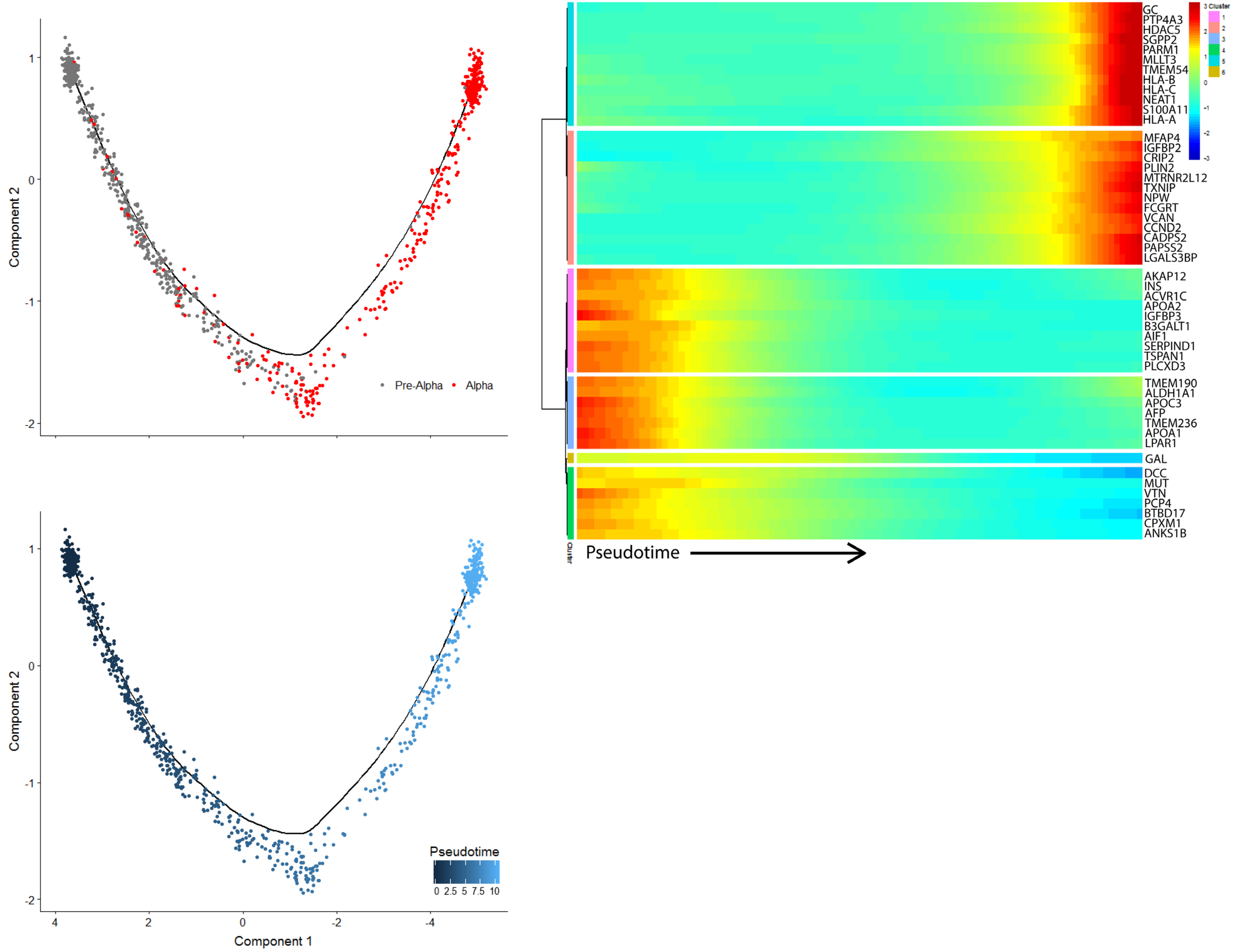

Supplemental Figure 17

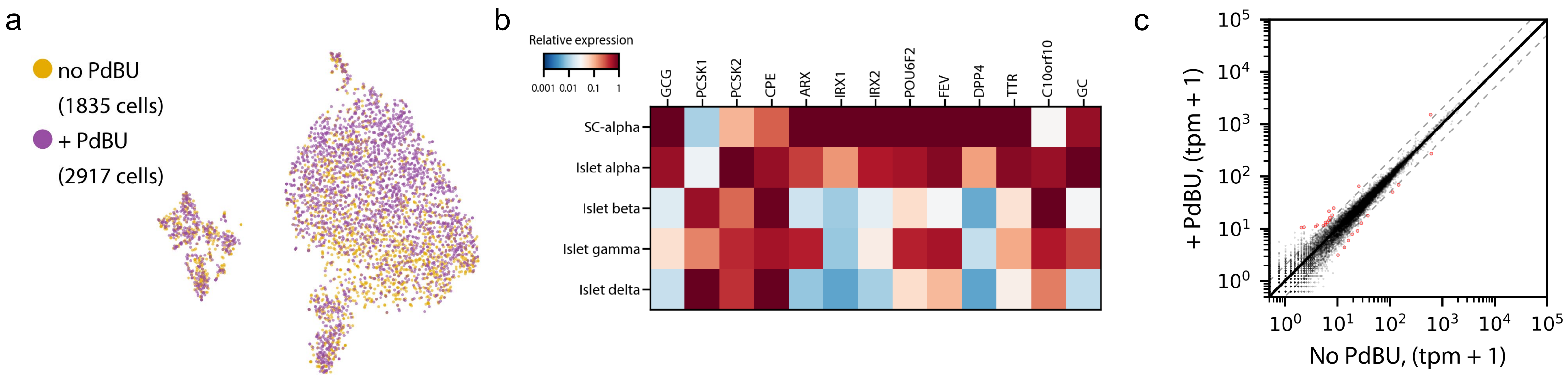

Supplementary Figure 17. Single cell RNAseq analysis of cells treated with and without PDBu. a) tSNE plot of cells differentiated with PDBu and without PDBu demonstrated no significant difference in gene signature despite specific differences in processing and expression of insulin and glucagon. b) Heatmap of key alpha cell marker transcript expression levels in SC-alpha cells compared to endocrine cell types from human islets. (Islet data obtained from Baron et al[23]) c) Differential expression analysis of pre-alpha cells and SC-alpha cells derived in the presence and absence of PDBu. Differentially expressed genes are marked in red and are listed in Supplemental Table 2.

Supplemental Figure 18

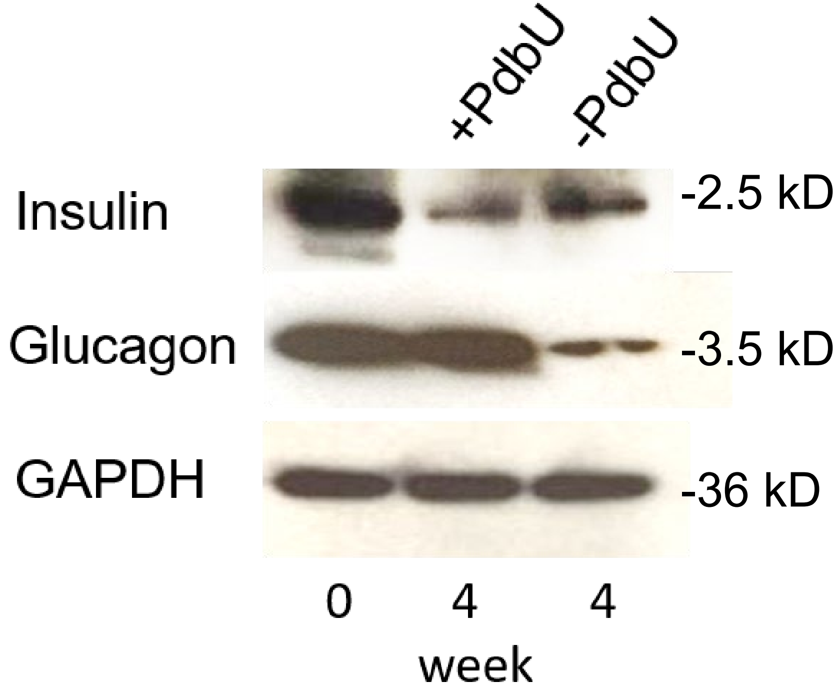

Supplementary Figure 18. Western blot analysis to show insulin and glucagon protein change in stage 6 cells treated with and without PDBu

Supplemental Figure 19

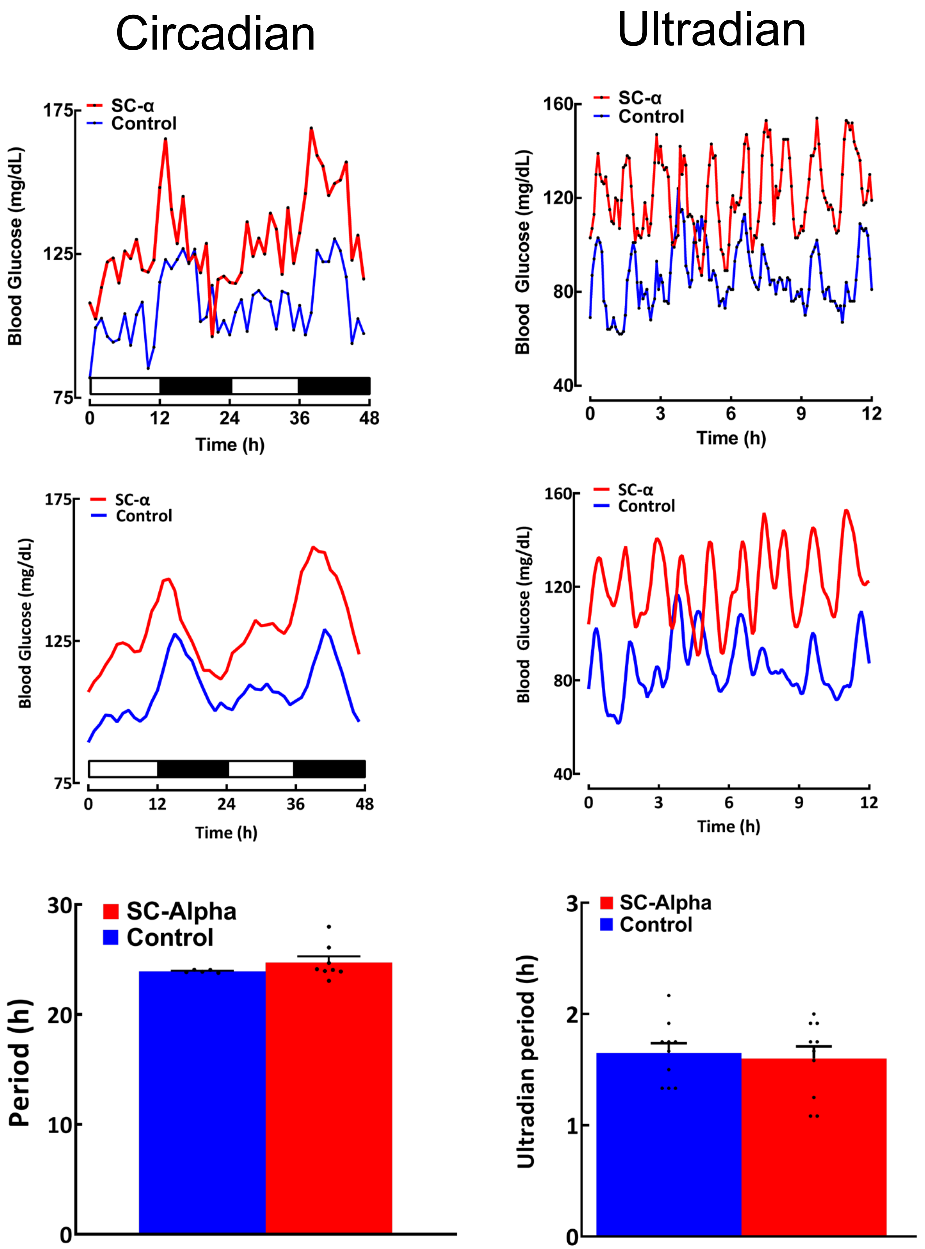

Supplemental Figure 19. Analysis of continuous glucose monitoring data from mice transplanted with SC-alpha cells or control animals during normal feeding and activity. Blood glucose concentration is maintained upon transplantation of SC-alpha cells as shown by raw (top) and smoothed (middle) averages of blood glucose for each cohort. Circadian rhythmicity of glucose was assessed in animals with a significantly dominant circadian period (n=5 control, n=8 SC-Alpha). Ultradian rhythmicity of glucose was assessed in animals with a significantly dominant ultradian period (n=10 control and SC-Alpha). Data is presented as mean ± SEM. The circadian and ultradian periodicity (bottom) is not significantly modified upon transplantation of SC-alpha cells.

Supplemental Figure 20

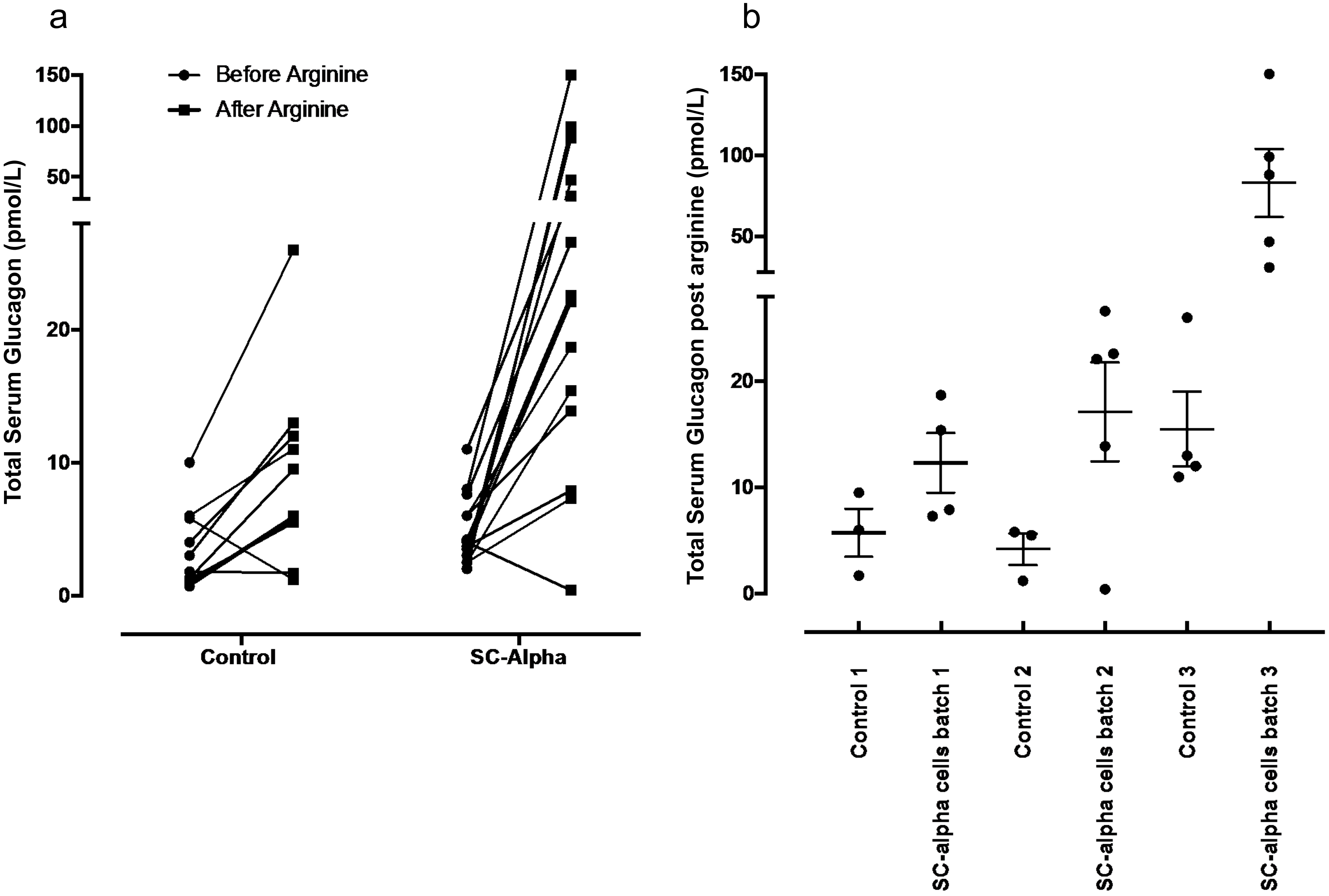

Supplemental Figure 20. Serum glucagon levels in response to an arginine bolus for 3 batches of SC-alpha cells generated in the 1016 iPS cell line at 4 weeks post-transplant. SC-alpha transplanted mice (n=5 animals) and control animals (n=5 animals) were fed ad libitum and injected i.v. with arginine. Serum glucagon levels were measured at time 0 and 1 minute post injection. a) Control and SC-alpha transplanted animals before and after arginine injection demonstrate significant increases in serum glucagon concentrations after arginine injection. b) comparison of serum glucagon for each batch of SC-alpha cells post arginine. Transplanted animals show an elevated level of serum glucagon. Bars represent the mean +/- SEM.

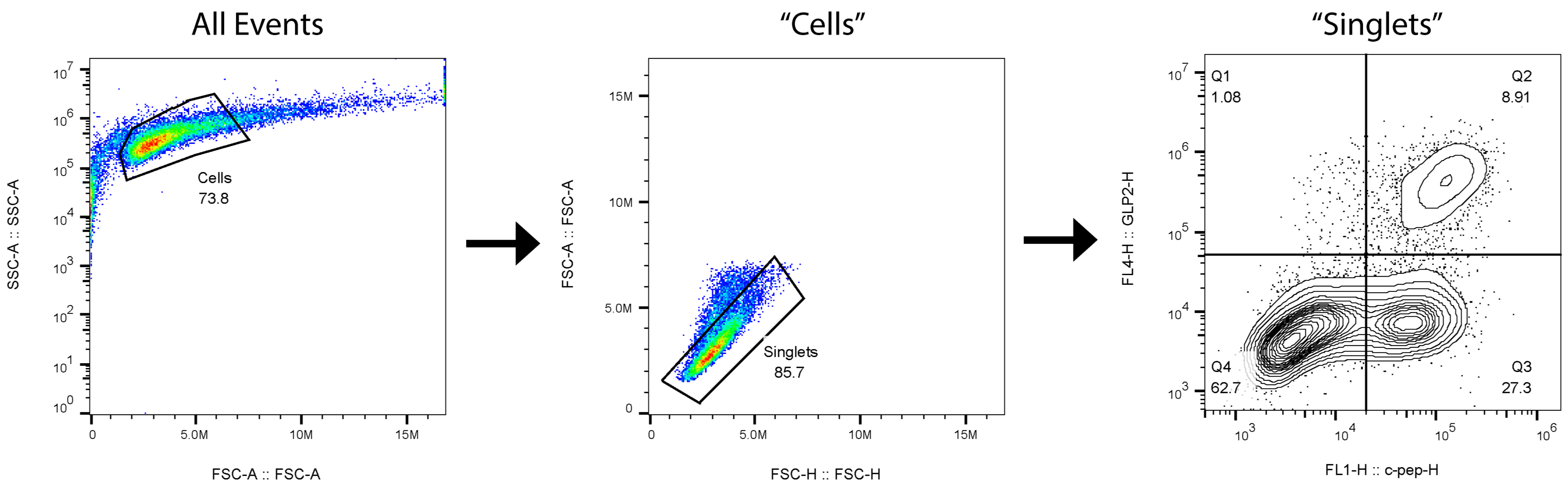

Supplemental Figure 21. Flow cytometry gating strategy for all flow cytometry shown in both main figures and supplementary information. All captured events are first gated by forward scatter area, side scatter area. Doublet discrimination is performed to define all singlets by gating along the diagonal of forward scatter height, forward scatter width. Final flow cytometry plots are shown as contour plots, with outliers shown.

Supplemental Figure 2c

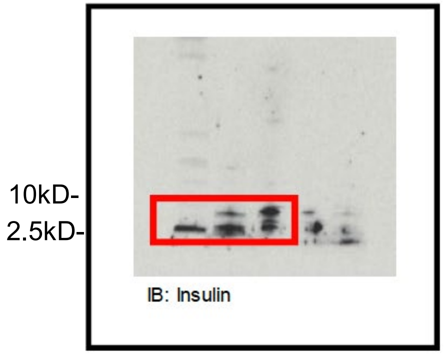

Supplemental Figure 18

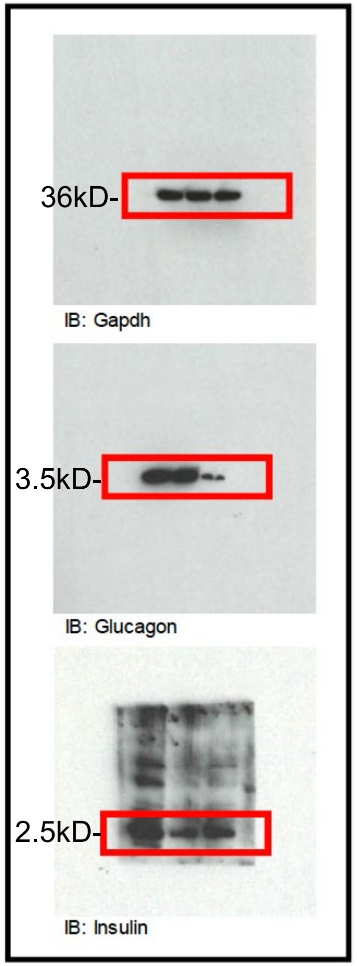

Supplemental Figure 22. Full Western blot images for supplemental figures S2C and S18. Raw scans with cropping lines marked in red.

Supplemental Table 1

Chemical Name	Final concentration	Target/Action	Vendor & Catalog Number
Rho Kinase Inh, Y-27632 (Rhoki)	10 μM	Rho Kinase Inhibitor	DNSK, DNSK-KI15-02
Activin A	100 ng/mL	Smad activation and regulation of activin-responsive gene transcription.	R&D Systems, 338-AC
Chir-99021 (Chir)	3 μM	GSK-3α/β inhibitor	TOCRIS, 4423
Keratinocyte Growth Factor (KGF)	50 ng/mL	FGFR2b agonist	PEPROTECH, 100-19
Retinoic Acid (RA)	2 μM	RAR a,b,g agonist	Sigma Aldrich, R2625
LDN193189 Hydrochloride (LDN)	200 nM	BMP type 1 receptor inhibitor	Sigma Aldrich, SML0559
Alk5 Inhibitor II (Alk5i)	10 μM	Alk5 inhibitor - inhibits TGF-β/Activin signaling	DNSK, DNSK-ALK5-02
Phorbol-12,13-dibutyrate (PDBu)	500 nM	PKC Activator	Millipore-Sigma, 524390

Supplemental Table 1. Factor concentrations used during the differentiation protocol (Stage1-6).

Supplemental Table 2

	+ PDBu	No PDBu
LGI2	10.467644	2.060103
CH25H	10.623878	2.317616
KCNJ8	21.560223	6.952848
MOB3B	24.684893	8.497925
RABEPK	11.717512	4.120206
TCF24	10.936345	3.862693
IGFBP2	1512.027792	602.322612
ELMO1	64.524435	25.751287
DACT2	18.123086	7.982899
MAPT	16.092050	7.210360
CDR2	13.123614	5.922796
PLTP	11.405045	5.407770
NR4A1	14.373482	6.952848
LY75	13.279847	6.437822
FAM43A	11.873746	5.922796
GUCY1A2	17.654385	38.369418
GRM1	12.811147	28.841422
DPF1	7.811675	19.313466
FXYD3	68.430272	147.297364
PLCXD2	5.936873	16.223311
ALOX5AP	3.124670	10.300515
AFP	49.994719	113.820690
INS	271.221352	620.863539
FOSB	9.061543	24.206210
RASSF6	4.374538	13.648182

Supplemental Table 2. Differentially expressed transcripts between SC-alpha cells derived with and without PDBu treatment.

Supplemental Table 3

Chemical Name	Final Concentration	Action/Target	Vendor	Catalog Number
CHIR99021	3 uM	Glycogen synthase kinase 3 beta (GSK-3β) inhibitor	Stemgent	04-004
Dorsomorphin	500 nM	AMPK Inhibitor	Calbiochem	171261
PD0325901	500 nM	Mitogen-activated protein kinase inhibitor	Stemgent	04-0006
IWP3	500 nM	Inhibitor of Wnt Production	Cayman Chemical	13953
AA (Activin A)	100 ng/mL	Member of the TGF-beta superfamily	R&D Systems	338-AC
LDN-193189	200 nM	ALK2 and ALK3 inhibitor	Stemgent	04-0074
PD173074	500 nM	Tyrosine kinase inhibitor	Stemcell Technologies	PD173074
KGF (FGF7)	50 ng/mL	KGF (FGF-7) signals through FGFR 2b	PeproTech	100-19
Gefitinib	500 nM	EGFR-tyrosine kinase inhibitor	Santa Cruz Biotechnology	SC-202166
SF1670	500 nM	PTEN inhibitor	Cellagen Technologies	C7316
AICAR	500 nM	AMPK activator	Tocris	2840
Sant-1	250 nM	Inhibitor of hedgehog signaling	R&D Systems	1974
LY411575	500 nM	Gamma secretase inhibitor	Stemgent	04-0054
Shz-1	500 nM	Activator of early cardiac genes in pluripotent stem cells	Stemcell Technologies	73422
RG108	500 nM	Non-nucleoside DNA methyltransferase inhibitor	Calbiochem	260920
Purmorphamine	500 nM	Smoothened (Smo) receptor agonist	Enzo Life Sciences	ALX-420-045-M005
IBMX	500 nM	PDE inhibitor (non-selective)	Stemcell Technologies	72762
PDBu (Phorbol 12,13-dibutyrate)	500 nM	Protein kinase C activator	EMD	524390
BIX01294	500 nM	GLP and G9a inhibitor	Tocris	3364
Reversine	500 nM	Aurora A, B, and C kinase inhibitor	Cayman Chemical	10004412
LE135	500 nM	Selective RARβ antagonist	Tocris	2021
All Trans Retinoic Acid	2 uM	Binds to retinoic acid receptor	Enzo Life Sciences	BML-GR100-0500

Chemical Name	Final Concentration	Action/Target	Vendor	Catalog Number
Sunitinib, Free Base	500 nM	An inhibitor of receptor tyrosine kinases	LC Laboratories	S-8877
Stauprimide	500 nM	Inhibits NME2 nuclear translocation	Sigma -Aldrich	S2951
AG556	500 nM	EGFR-kinase inhibitor	Tocris	0616
XXI (Compound E)	1 uM	Gamma secretase inhibitor	Enzo Life Sciences	270-415-C250
BMS 777607	500 nM	c-Met inhibitor	Selleck	S1561
SB431542	500 nM	Selective inhibitor of TGF-βRI, ALK4 and ALK7	Stemgent	04-0010
PD169316	500 nM	Selective inhibitor of p38 MAPK	Cayman Chemical	10006727
ALK5 Inhibitor II	10 uM	TGF-β inhibitor	Stemgent	04-0016
ARQ-197	500 nM	c-Met inhibitor	Active Biochem	A-1109
U0126	500 nM	Selective inhibitor of MEK1 and MEK2	Cayman Chemical	70970
T3 (Triiodothyronine)	1 uM	Thyroid hormone	EMD	64245
CP 690550	500 nM	JAK3 inhibitor	Santa Cruz Biotechnology	SC-207457
MS275	500 nM	HDAC (Class I) inhibitor	Cayman Chemical	13284
TWS119	500 nM	GSK-3β inhibitor	Stemcell Technologies	73514
Betacellulin	20 ng/mL	Member of the EGF family of cytokines	R&D Systems	261-CE
INCB018424	500 nM	JAK1/2 inhibitor	Selleck	S1378
SU5402	500 nM	Potent FGFR and VEGFR inhibitor	Tocris	3300
DEAB	500 nM	Inhibitor of cytosolic (class 1) aldehyde dehydrogenase (ALDH)	Sigma -Aldrich	D86256
SC1 (Pluripotin)	500 nM	Dual ERK1/RasGAP inhibitor	Santa Cruz Biotechnology	SC-255607
A769662	500 nM	Potent AMPK activator	Stemcell Technologies	72922
GABA	500 nM	Activator of GABA Receptor.	Santa Cruz Biotechnology	sc-203053
DMSO	0.1%	Control	Fisher	BP231

Supplemental Table 3. Chemicals and concentrations used for the small molecule screen.
